# CRISPhieRmix: a hierarchical mixture model for CRISPR pooled screens

**DOI:** 10.1186/s13059-018-1538-6

**Published:** 2018-10-08

**Authors:** Timothy P. Daley, Zhixiang Lin, Xueqiu Lin, Yanxia Liu, Wing Hung Wong, Lei S. Qi

**Affiliations:** 1 Department of Statistics, Stanford University, 450 Serra Mall, Stanford, 94305 USA; 2 Department of Bioengineering, Stanford University, 443 Via Ortega, Stanford, 94305 USA; 3 Department of Chemical and Systems Biology, Stanford University, 443 Via Ortega, Stanford, 94305 USA; 4 ChEM-H Institute, Stanford University, 443 Via Ortega, Stanford, 94305 USA; 5 Department of Biomedical Data Science, Stanford University, 1265 Welch Road, Stanford, 94305 USA; 6 Present Address: Department of Statistics, The Chinese University of Hong Kong, Shatin, N.T., Hong Kong SAR, China

**Keywords:** CRISPR screen, CRISPR interference, CRISPR activation, sgRNA design, Mixture models, Local fdr

## Abstract

Pooled CRISPR screens allow researchers to interrogate genetic causes of complex phenotypes at the genome-wide scale and promise higher specificity and sensitivity compared to competing technologies. Unfortunately, two problems exist, particularly for CRISPRi/a screens: variability in guide efficiency and large rare off-target effects. We present a method, CRISPhieRmix, that resolves these issues by using a hierarchical mixture model with a broad-tailed null distribution. We show that CRISPhieRmix allows for more accurate and powerful inferences in large-scale pooled CRISPRi/a screens. We discuss key issues in the analysis and design of screens, particularly the number of guides needed for faithful full discovery.

**Electronic supplementary material:**

The online version of this article (10.1186/s13059-018-1538-6) contains supplementary material, which is available to authorized users.

## Background

CRISPR interference and CRISPR activation (CRISPRi and CRISPRa, respectively) pooled screens allow for high-throughput functional interrogation of genes through modifications in gene expression [1–3]. Variable levels of inhibition and activation allow for the investigation of a broad range of expression changes [4, 5]. This is in contrast to CRISPR knockout (CRISPRko) screens, where ablation typically results in gene silencing [6, 7]. CRISPRa and CRISPRi screens have multiple other benefits relative to CRISPRko. Both allow for the investigation of a broader class of genomic elements, such as long non-coding RNAs (lncRNAs) [4] and enhancer elements [8, 9]; CRISPRa specifically allows for gain of function investigations, such as targeted differentiation and trans-differentiation of specific cell types [5]; and CRISPRi does not generate double-stranded breaks that can induce a gene-independent DNA damage response that can lead to cell death [10, 11].

Unfortunately, the variability in inhibition and activation levels creates issues in the analysis and identification of genes associated with the phenotype under study. Chromatin organization and epigenetic effects such as nucleosome positioning [12], DNA folding [13, 14], and variable transcription start site usage [15] can lead to unpredictable and irregular guide efficacy. Even for CRISPRko, varying insertion size and allele diversity can result in variable effects across sgRNAs [16]. This results in a broad range of sgRNA effects and, more critically for CRISPRi/a experiments, a percentage of sgRNAs that have no effect, even for true positive genes. In addition, despite promises of high specificity of CRISPR technologies, we tend to observe rare but large off-target effects in all three types of screens. These issues make it problematic to systematically score genes to determine which genes are highly likely to contribute to the phenotype of interest, and deserve further investigation.

**Fig. 1:**
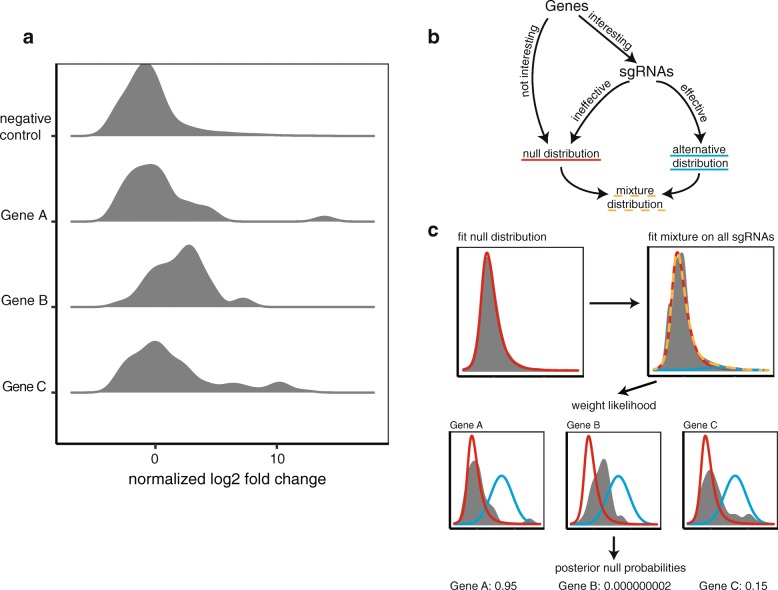
**a** A hypothetical situation. Gene A has a single sgRNA with a large effect, while the rest of the sgRNAs have little effect. Gene B has very few sgRNAs with large effects, but the distribution looks shifted from the negative control. Gene C appears to follow a clear mixture distribution, with some percentage of the sgRNAs having a large effect but the majority looking like the negative control distribution. **b** The hierarchical structure of CRISPhieRmix. Genes can either have no association with the phenotype, in which case they are not interesting, or they are interesting and deserve further investigation. Without prior knowledge, the genes can be considered as arising from a mixture distribution. For genes that are interesting, we model sgRNAs as a mixture distribution as well, with some sgRNAs having no effect and following the null distribution and the others having some effect and following the alternative distribution. **c** Overview of the CRISPhieRmix algorithm. First, a broad-tailed null distribution is fit using negative control sgRNAs. Then, a mixture distribution is fit on all sgRNAs, ignoring gene identities. Finally, for each gene, CRISPhieRmix uses the mixture distribution to calculate the local false discovery rate, equal to the posterior probability that the gene is null. Of the three genes in **a**, we estimate that gene A is highly likely to be null, gene B is highly likely to be a true hit, and gene C is probably non-null

Consider the situation shown in Fig. 1a. Gene A has a single sgRNA with an extremely large effect, and the remainder seem to closely mirror the distribution of the negative control sgRNAs. It is difficult to determine if gene A is negative, and this is a random off-target effect or, alternatively, gene A is positive and by random chance only one guide worked. Gene B, on the other hand, has no guides with such a strong effect, but the majority of the distribution is shifted away from the negative control guides. This indicates that gene B has a small but consistent effect for the phenotype. Finally, gene C follows a clear mixture distribution with a majority of the guides having no effect and a clear percentage having some effect, but all with a lower effect than the top guide from gene A. Under some strategies of ranking guides, such as looking at only the few strongest sgRNAs for each gene, gene A might be ranked the highest of the three while gene B might be missed. Alternatively, if the analysis fails to take into account the mixture assumption, then gene A will be ranked low but gene C might be missed because the majority of the guides support a null phenotype. How can we suitably weigh the evidence for genes A, B, and C? If prior knowledge is available, e.g., gene A is unlikely to be related to the phenotype under investigation, then we may attribute the large effect of a single guide to an off-target effect. But without prior knowledge, how can we do this in a systematic manner?

Existing methods for analyzing pooled CRISPR screens typically rely upon a hypothesis testing paradigm. The observed changes in abundance are tested against a null distribution of changes, and global false discovery rates (FDRs) are computed using the estimated *p* values. However, departure from the assumed model can cause both false discoveries and false negatives, resulting in a decreased true positive rate (TPR) and an increased FDR. To solve these issues, we take a mixture deconvolution approach to estimate local false discovery rates, similar to the approaches of Efron [17], McLachlan et al. [18], and Strimmer [19] in analyzing gene expression data. To account for variable guide efficiency, we use a hierarchical mixture model (Fig. 1b), and to account for possible off-target effects, we use a broad-tailed distribution that is estimated from negative control guides (Fig. 1c). This allows for more flexibility in the model and, when guide effects are variable, both decreases the false discovery rate and increases the true positive rate on simulated data. We tested our method on the identification of essential genes from a previous CRISPRi screen and found that our method identifies many more essential genes than other methods, with many of those missed showing a clearer mixture distribution than the genes found by all methods. We also tested CRISPhieRmix on CRISPRko screens for essential genes and found that CRISPhieRmix is competitive with the current state of the art algorithms, despite the fact that there is less variability between sgRNAs targeting the same gene. Finally, we investigate the performance of our method as a function of the number of sgRNAs per gene and proportion of effective sgRNAs. Our results suggest that current studies commonly use too few sgRNAs for full discovery.

## Results

### Hierarchical mixture model

Suppose that *G* genes or features (typically genes and we will refer to them generally as genes from here on) are interrogated with *N* sgRNAs or guides (we will use the two terms interchangeably) in a CRISPR screen. Following the terminology of Efron [20], the goal of the screen is to identify the class of “interesting” genes that are likely to be causal for the phenotype studied and are candidates for further investigation. We assume that the vast majority of genes are uninteresting or null. The observed changes in the abundance of the guides corresponding to the uninteresting genes should all follow some distribution *f*_0_ that we call the null distribution. Of course it may be possible that a large number of genes have small effects, but these are of little interest to expend further resources investigating and are grouped with the uninteresting genes.

We assume that changes in abundance of the guides corresponding to interesting genes follow an alternative distribution *f*_1_ that is sufficiently different from *f*_0_. Without prior knowledge of which genes are interesting or uninteresting, the observed changes in guide abundances will follow a mixture distribution (1−*p*)*f*_0_+*pf*_1_, with *p* equal to the proportion of interesting genes. This is commonly called the two groups model in the statistics literature [21].

When we examine positive hits, for example at known essential genes in CRISPRi dropout screens [22, 23], we observed a clear mixture distribution on guides targeting genes known to be interesting, essential in this case (Additional file 1: Figure S1). Similarly, we observe a mixture distribution in known interesting genes CRISPRko screens, albeit to a lesser extent (Additional file 1: Figure S1). To handle the possibility that some guides are ineffective, we assume that the guides from interesting genes also follow a mixture distribution. Specifically, we assume that some percentage 1−*q* of guides will “not work” and follow the null distribution *f*_0_, while the remainder will follow the alternative distribution *f*_1_.

The full model is detailed in the “Model” section. One issue in estimating the model is that *p* and *q* are not simultaneously identifiable. Since our primary interest is in gene-level inference, we integrate out the nuisance parameter *q* (“Identifiability” section). For each gene, we compute the posterior probability that it is null, called the local false discovery rate (local fdr) by Efron [17, 20]. To allow for comparison with other methods that use a hypothesis testing paradigm, we compute global false discovery rates (global FDR) by averaging the empirical distribution of local false discovery rates (“The local false discovery rate” section).

### Normal hierarchical mixture

Consider the case where both *f*_0_ and *f*_1_ are normal distributions. As we will discuss later, this is likely an incorrect assumption, but this simple case will illustrate the power of the hierarchical mixture.

Evers et al. [22] produced a “gold standard” dataset, performing both a CRISPRko screen and a CRISPRi screen for 46 known essential genes and 47 known non-essential genes. This dataset had an average of 7.3 guides per gene with 3 replicates, each sequenced to an average of 4500 reads per guide in every experiment. Therefore, sequencing depth and variability between replicates are unlikely to introduce bias and create difficulties in the analysis, at least compared to other data sets that typically have 2 replicates and sequence 100 to 1000 reads per guide per experiment. We used DESeq2 [24] to compute sgRNA-level normalized log2 fold changes for this CRISPRi screen (Additional file 1: Figure S1).

We applied the normal hierarchical mixture (NormHierMix) to the estimated log2 fold changes, as well as MAGeCK in both robust ranking aggregation (RRA) [25] and maximum likelihood estimation (MLE) [26] mode to the raw count data. We measure how well the methods distinguish essential from non-essential genes by the area under the receiver operator characteristic curve (ROC-AUC). The ROC-AUC of NormHierMix was much higher than either MAGeCK methods (Additional file 1: Figure S2), indicating that it cleanly discriminated essential from non-essential genes better than either MAGeCK methods.

We estimated the global FDR of NormHierMix by averaging all smaller local fdrs. Because there are so few genes in this screen, this estimate may be highly variable. As a check, the maximum global FDR estimated by NormHierMix is 0.44, not far from the fraction of genes that are non-essential (47/93 ≈ 0.51), and the latter is equal to the true FDR if all genes were called essential. At a global FDR of 0.1, NormHierMix called 51 genes as essential, with 42 correctly essential, giving an empirical FDR of 0.18. MAGeCK RRA only called 12 genes as essential (but at an empirical fdr of 0), and MAGeCK MLE called a single gene as essential. It appears that both MAGeCK methods are far too restrictive in calling genes, possibly because of the lack of a mixture assumption that is needed for CRISPRi/a screening data, but the normal hierarchical mixture model is too liberal, that we believe is due to the fact that the null guide distribution is not normal.

### Choice of the null distribution *f*_0_

Under proper normalization, *f*_0_ should be standard normal [20]. In our experience, we typically observe that the distribution of non-targeting negative control sgRNAs, which should reflect the null distribution, has longer tails than a normal distribution (Additional file 1: Figure S1). We find that the normal distribution provides a poor fit for the empirical distribution of both truly negative genes (Additional file 1: Figure S1) and negative control guides (Additional file 1: Figure S3).

While some screens investigate bidirectional phenotypes, e.g., ricin resistance and susceptibility in Gilbert et al. [2], most screen for one direction, e.g., screens for essential genes look for depleted genes [6, 27] and gain of function screens look for enriched genes [5]. We typically see a longer tail in the distribution of negative control guides in the same direction as the investigation. This makes it difficult to estimate the null distribution without negative control guides, as Efron [20] does in the case of gene expression data by using the central peak in the distribution with the reasonable assumption that all observations in the main peak are highly likely to be null. A broader tail towards one side means that the central peak is not likely to reflect the whole distribution. It is clear that we need a distribution that is both asymmetric and heavier tailed than the normal distribution. We will have to choose a family of distributions that is flexible enough to capture this behavior.

We tested several families of distributions, including non-parametric approaches, and found success with the skew-*t* distribution (“The skew-*t* distribution” section). The usage of a more general family of distributions improves the fit tremendously in all three types of CRISPR screens, with both visual checks (Additional file 1: Figures S3 & S5 and Additional file 1: Figures S4 & S6) as well as with model selection by Bayesian information criteria (BIC) (Table 1 and Additional file 1: Table S1). This gain in flexibility in modeling the null distribution will help us to down-weight genes that have a single outlier observation, like gene A in Fig. 1. In our experience, such genes are problematic to deal with systematically. They occur too frequently and are observed too consistently across experiments to be considered true outliers, yet they often make little sense biologically. Improving our understanding of how such outliers occur will help to improve experimental protocol and reduce their occurrence. In the meantime, our flexible statistical modeling strategy can help to handle such problems and improve the accuracy of inferences.

**Table 1:** Bayesian information criteria (BIC) of the normal model (shown in Fig. 3) and the skew-*t* fit (shown in Additional file 1: Figure S4) for the negative control guides of the indicated experiments

	Gilbert (2014)	Liu (2017)	Jost (2017)CRISPRi	Jost (2017) CRISPRa
Normal BIC	12771.8	1158.6	− 1366.1	4455.9
Skew-*t* BIC	*11135.2*	*− 431.9*	*− 4098*	*1011.2*

A smaller BIC is preferred and is in italics for each experiment

Note that permutation-based estimation of the null distribution, such as in Li et al. [26] and Jia et al. [28], should in theory handle such situations, as long as there is sufficient data to empirically estimate the null distribution.

### Comparison of methods on a semi-simulated dataset

To produce a dataset to compare methods using negative controls, we take a hybrid approach to simulation. The true positive genes are taken directly from the experiment, and negative genes will be simulated from negative control guides. Rosenbluh et al. [23] performed a CRISPRi screen on 33 genes with an average of 68 guides per gene targeting each TSS, as well as 959 negative control guides. These genes were chosen to be known essential genes or oncogenes. Since the screens were performed on cancer cell lines, silencing of these genes is expected to inhibit cell growth or lead to cell death and should decrease the abundance of the gene targeting guides in either case. Unfortunately, the authors did not include any negative control genes, genes that are known to be not essential or affect the growth rate, so we will simulate them from the negative control guides.

To simulate null non-essential genes, we first simulated the number of guides per gene by sampling from a negative binomial distribution with parameters fit from the essential gene counts (*μ*=67.8, *r*=26.3). We then sampled a negative control guide at random and computed the simulated counts as independent negative binomial random variables with mean parameter equal to the observed counts for the sampled negative guide and the size parameter equal to 200. We did this for 104 (=3·34) negative genes, so that *p*=0.25. This gave us 5920 guides corresponding to negative genes. The distribution of DESeq2-normalized log2 fold changes are shown in Additional file 1: Figure S7. The guides corresponding to the simulated negative genes had a very similar empirical distribution as the negative control guides. The guides targeting essential genes have a peak at zero, which looked similar to the distribution of negative control guides, but with a longer and heavier tail on the negative side. These are the guides that induce a phenotype that resulted in their dropout from the pool of cells.

We applied CRISPhieRmix to this simulated data (the fit is shown in Additional file 1: Figure S8). At a local fdr of 0.2 (or a posterior probability equal to 0.8 of begin essential), we identified 18 genes (Additional file 1: Figure S9), all of which are truly essential. These 18 genes are assigned a very high probability of being interesting, and the other 16 true positive genes are assigned a lower probability. An inspection of the distribution of each class reveals a clear difference in the distribution of these two groups (Additional file 1: Figure S10). At a global FDR of 0.1 (as estimated by averaging the ordered local fdr values), CRISPhieRmix identified 21 genes as interesting with all correct, and at a global FDR of 0.2, CRISPhieRmix identified 26 genes as interesting with 24 correct, giving an empirical FDR of 0.08.

For comparison, we also applied MAGeCK MLE and a Mann-Whitney test that compares the log2 fold changes of gene targeting guides versus the negative control guides. For the Mann-Whitney test, we used Benjamani-Hochberg-corrected false discovery rates. We also applied MAGeCK RRA to the count data, but no genes were determined to be significant at any FDR level, so the results are excluded from further analysis.

**Fig. 2:**
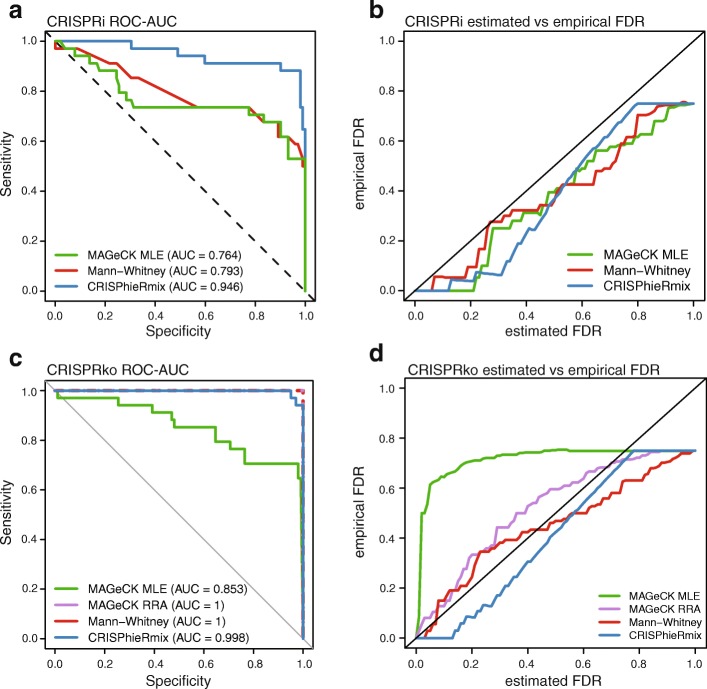
**a** Receiver operator curves for gene scores computed by CRISPhieRmix, the Mann-Whitney test, and MAGeCK MLE on the Rosenbluh CRISPRi simulated data. The diagonal line *y*=1−*x* indicates performance by random chance. **b** The corresponding estimated false discovery rates (*x*-axis) plotted against the corresponding empirical false discovery rates (*y*-axis). The diagonal line *y*=*x* indicates perfect control of the false discovery rate. **c** and **d** are the same as **a** and **b**, respectively, but on the Rosenbluh CRISPRko simulated data

We found that both CRISPhieRmix highly discriminated the positive genes from the simulated negative genes, with an area under the receiver operator curve (ROC-AUC) of around 0.95 (Fig. 2a). The other tested methods had significantly lower ROC-AUCs, indicating that CRISPhieRmix can better discriminate the true genes from the simulated negative genes.

The ROC-AUC only takes into account the gene ranking and ignores the actual threshold used in the gene rankings. We also investigated which algorithms correctly control the false discovery rates at their stated values (Fig. 2b). In this case, all methods control the FDR properly, with curves near or below the diagonal line which indicates perfect estimation of the FDR. Note that this is not the case for the normal hierarchical mixture model (Additional file 1: Figure S11), as the normal distribution is unable to handle what is commonly considered outlier effects.

We performed identical simulations with the corresponding CRISPRko data from Rosenbluh et al. [23]. In this case, MAGeCK RRA obtained usable results, so we included it in our comparisons. MAGeCK RRA and the Mann-Whitney test were able to perfectly distinguish the true genes from the simulated null genes, as indicated by an ROC-AUC of 1 (Fig. 2c). CRISPhieRmix made one mistake, ranking only one null gene above only one true gene, resulting in an ROC-AUC of 0.998. CRISPhieRmix was able to do this while controlling the empirical FDR, something all other methods fail to do (Fig. 2d). The stark difference in performance for the Mann-Whitney test between the CRISPRi and the CRISPRko simulations is interesting, and we believe it may be due to the differences in guide efficiency between the two technologies. We discuss this in more depth in the “Discussion” section.

Overall, these results indicate that CRISPhieRmix can distinguish true positive genes from null genes while effectively controlling the false discovery rate and can perform well on both CRISPRi and CRISPRko screens.

### Performance with fewer guides

The Rosenbluh data has a lot of guides per gene, and this helps to distinguish the true genes from the simulated null genes. Most libraries have fewer sgRNAs per genes, typically 5. To evaluate the performance of the methods when fewer guides are available, we randomly downsampled the simulated CRISPRi and CRISPRko libraries 100 times at various levels of average number of sgRNAs per gene, from a lower limit of 5 to the total number of guides per gene (68 in the CRISPRi simulated data and 59 in the CRISPRko simulated data). We looked at the ROC-AUC, the percentage of true essential genes called at an estimated FDR of 0.1 (TPR), the empirical FDR of at the same cutoff, and running times for CRISPhieRmix, MAGeCK MLE, and Mann-Whitney tests in the CRISPRi simulated data. For the CRISPRko simulated data, we also included MAGeCK RRA in our comparisons.

**Fig. 3:**
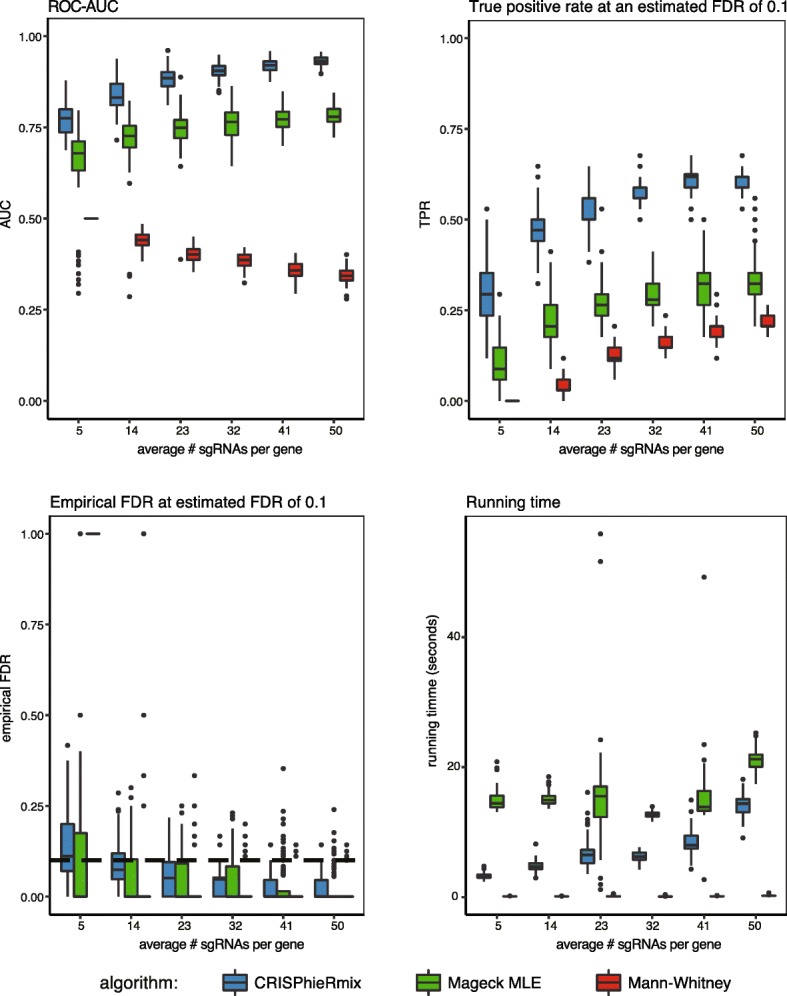
A comparison of algorithms as a function of sgRNAs per gene in the simulated CRISPRi screen. The area under the receiver operator curves (AUC), true positive rate (TPR) at a threshold of 0.1 global false discovery rate (FDR), empirical FDR at an estimated FDR of 0.1, and running times of all algorithms are shown. The dashed line indicates an FDR of 0.1, or perfect control of the FDR

For the CRISPRi downsample simulations, we observed that the number of truly essential genes called by CRISPhieRmix, as well as the empirical FDR and the ROC-AUC, stayed relatively stable when we downsampled down to an average of of 23 guides per gene (Fig. 3). Beyond that, the performance dipped slightly at 14 and 5 guides per gene, but at 5 guides per gene, CRISPhieRmix loses the ability the control the FDR, albeit slightly. MAGeCK MLE, in contrast, controlled the FDR at all number of guides, but the ROC-AUC and TPR were lower than that of CRISPhieRmix at all numbers of average guides per gene. In contrast, the performance of the Mann-Whitney tests decreased sharply at any level of downsampling. This agrees with the results that we present in the next section, as there we have 10 guides per gene.

For the CRISPRko downsample simulations (Additional file 1: Figure S12), MAGeCK RRA had a nearly perfect ROC-AUC for all levels of downsampling, with CRISPhieRmix close behind. Similarly, MAGeCK RRA had the highest TPR at all levels of downsampling, at a cost of not quite controlling FDR. CRISPhieRmix, on the other hand, perfectly controls the FDR up till 5 guides per gene, but at the cost of lower discovery in the true positive rate. Meanwhile, the Mann-Whitney test was competitive for large numbers of sgRNAs per gene, but broke down for the lowest numbers. These results indicate that MAGeCK RRA is still the current state of the art for analyzing CRISPRko screens. CRISPhieRmix still performed well in these simulations, but our belief is that the signal from CRISPRko screens is typically strong enough that there will be a loss of power when including a mixture assumption. It is in cases where one can expect variable sgRNA efficiency or effects that we believe CRISPhieRmix will perform the best.

### CRISPhieRmix increases the identification of truly essential genes in dropout screens

In previous existing datasets, the ground truth is not known. This forces us to use proxy measures to compare and benchmark methods. In the previous section, we used a semi-simulated approach, but the assumptions in such simulations (such as the assumption that the distribution of negative control guides and negative gene targeting guides is the same) may not reflect the underlying reality. In this section, we will use an experiment with a well-studied phenotype to compare and benchmark methods.

Gilbert et al. [2] performed a genome-scale CRISPRi screen to examine ricin sensitivity and resistance for 15,977 genes with 10 sgRNAs per gene and 10,569 negative control sgRNAs. They sequenced the initial population, the treated population, and the untreated population. To estimate ricin sensitivity, they compared the treated to the untreated population, as this will control for growth-related effects from the inhibited genes. We can, however, compare the untreated versus the initial population and look for genes that dropout of the screen. These genes should be responsible for either cell death or decreased cell growth. Thus, we can look at known essential and non-essential genes to compare various algorithms. Of course, essential genes vary by context and in strength. To prevent such bias, we will use the reference set of Hart et al. [29], who used multiple screens to find a core set of essential genes. We use their ConstitutiveCoreEssential gene set as a positive set of known essential genes and their NonEssential gene set as a negative set of known non-essential genes. The ConstitutiveCoreEssential gene set contains 217 genes, all interrogated by Gilbert et al. [2], and the NonEssential gene set contains 927 genes, with 460 interrogated by Gilbert et al. [2]. Naturally, since the cell type used in the experiment is a cancer cell line, we should expect a multitude of other types of genes to be positive in the screen, such as oncogenes or tumor suppressor genes. The reference set provides a known basis that we can use as a proxy to measure the performance of the tested methods.

We applied all the methods described in the “Comparison of methods on a semi-simulated dataset” section to the raw count data, computing log2 fold changes with DESeq2. The lowest *p* value calculated by MAGeCK RRA was 0.5 and identified no genes as significant at any FDR < 1, and we will not discuss those results any further.

At a global FDR of 0.1, CRISPhieRmix (fit is shown in Additional file 1: Figure S13) identified a total of 1425 genes as interesting with 172 of these contained in the ConstitutiveCoreEssential gene set and only 2 contained in the NonEssential set (Table 2), giving an estimated FDR of 0.01. At the same global FDR of 0.1, MAGeCK MLE identified 795 genes with 106 contained in the ConstitutiveCoreEssential gene set and 3 in the NonEssential set. This gives an empirical FDR of 0.028, indicating that MAGeCK MLE does a good job at controlling the FDR in this situation. The Mann-Whitney test, on the other hand, had difficulty in separating the two classes and does little better than random chance in distinguishing essential from non-essential genes (Table 2 and Additional file 1: Figure S14).

**Table 2:** The number of genes identified at a global FDR of 0.1. Essential genes are defined as genes in the ConstitutiveCoreEssential gene set of Hart et al. [29] and similarly for non-essential genes

	CRISPhieRmix	MAGeCK MLE	M-W
Total genes	1425	795	1383
Essential genes	172	106	52
Non-essential genes	2	3	96

*M-W* Mann-Whitney

MAGeCK MLE does a reasonably good job in discriminating essential from non-essential genes, but misses a lot of genes compared to CRISPhieRmix at the same FDR. In fact, CRISPhieRmix identifies nearly all (105 out of 106) of the genes identified by MAGeCK MLE. We examined the distribution of the genes called by both methods and the genes only called by CRISPhieRmix (Additional file 1: Figure S15). The essential genes identified only by CRISPhieRmix look much more like a mixture distribution with more guides that look null and weaker signal than the genes called by both methods. We verified this is true by looking at individual genes (e.g., Additional file 1: Figure S16). This indicates that MAGeCK needs more guides significantly different from the null distribution to identify genes. This is, of course, solved by the mixture assumption on guides in CRISPhieRmix.

We next sought to evaluate all methods on a CRISPRko screen, again using a dropout screen and evaluating methods based on the reference set of Hart et al. [29]. We applied CRISPhieRmix, MAGeCK MLE, MAGeCK RRA, and the Mann-Whitney test on the six experiments from Hart et al. [30], downloaded from the TKO website [31]. DESeq2 log2 fold changes are shown in Additional file 1: Figure S17. We only tested the methods on the base library, which has an average of 5 sgRNAs per gene targeting 17,231 genes. We did not test BAGEL [32] because it uses the reference set as a baseline in their semi-supervised method and would likely perform perfectly. Additionally, we are primarily interested in unsupervised identification of genes, as formulated in the two groups model [21]. This ensures the broadest applicability of the method, since even when there are known positive genes these can be used as a post-ranking validation.

In all six experiments, CRISPhieRmix and MAGeCK RRA performed the best, and actually quite similarly with a maximum difference in ROC-AUC of 0.02 (Additional file 1: Figure S18). Similarly, all methods performed similarly in controlling the FDR. This indicates that even on CRISPRko screens CRISPhieRmix can perform competitively with the current state of the art.

We used dropout screens to compare screens because more is known about the underlying biology of these screens than almost any other screen. The availability of a gold standard reference set gives us a proxy measure to easily compare methods. We should note, however, that we believe that dropout screens for essential screens have higher signal than other screens. When the signal is smaller, we believe that the mixture deconvolution approach of CRISPhieRmix will be able to better distinguish true genes from false positives.

### Designing experiments: how many guides to include to optimize discovery?

Recent works have developed methods to classify and design better guides that have higher on-target effects or, in other words, a higher chance of working [15, 23, 33]. Despite these improvements in guide design, it remains difficult to design guides with sufficiently strong functional phenotype (e.g., activate or repress genes to sufficient fold change for an observable phenotype). For example, if in a CRISPRa screen a gene is not primed for activation and its promoter is closed, then it might be difficult for the dCas9 to bind and sufficiently activate the target gene. Designing effective guides is a well-discussed issue in CRISPRko screens, see for example Listgarten et al. [34] and Haeussler et al. [35]. The difference in CRISPRko screens is that the chance that a random guide works is higher, but the libraries tend to have less guides per gene, typically 3 or 5 compared to 5 or 10 in CRISPRi/a screen. As such, there is still a small chance that no effective guides for a particular gene are in the library. Consider the case where 95% of guides work. If the library contains 5 guides per gene and we need at least 2 effective guides to prevent false positives, then the chance of missing a random gene is approximately 0.05%. On the other hand, if the library contains only 3 guides per gene, then the chance of missing a random gene is approximately 3%. Of course, this was just a hypothetical example but a real example was recently discussed in Rauscher et al. [36].

Rosenbluh et al. [23] estimated that only 40% of guides in their screen were effective at achieving transcriptional inhibition. To investigate the performance at other levels of guide efficiency, we will take a fully simulated approach. We will assume that the negative distribution is a skew-*t* with parameters *ξ*=0,*ω*=1,*α*=−1.5,*ν*=6; the positive genes have an effect size that is normally distributed with mean − 3 and standard deviation 0.75; and the effective guides are normally distributed with mean equal to the gene effect size and standard deviation equal to 1. This implies that the overall distribution of effective guides for positive genes is normal with mean − 3 and standard deviation 1.25. We varied the percentage of effective guides from 10 to 100% and varied the number of guides from 1 to 35. Example densities are shown in Additional file 1: Figure S19. We applied CRISPhieRmix and the Mann-Whitney test to the simulated data and looked at the percentage of positive genes correctly identified at an estimated FDR of 0.1 and the ROC-AUC, which measures how well the positive genes were ranked relative to null genes.

**Fig. 4:**
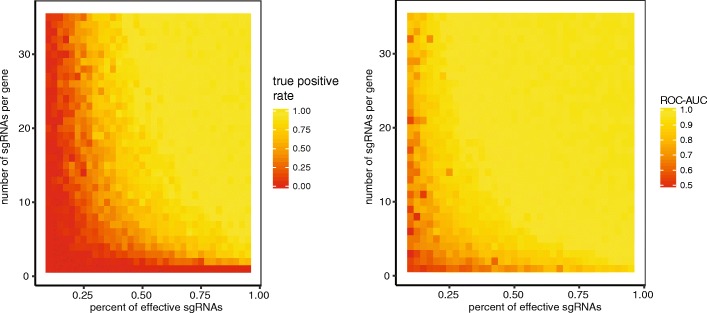
The true positive rate (the percentage of true interesting genes correctly identified) for CRISPhieRmix at an FDR of 0.1 (left) and ROC-AUC (right) for simulated data with varying percent of effective sgRNAs and number of sgRNAs per gene. The ROC-AUC is high for most parameters, implying that ranking the genes is usually easy, and the percent of correctly identified genes is much smaller, implying that it is difficult to correctly identify all truly interesting genes while simultaneously effectively controlling the false discovery rate

We found that even with a low number of guides (≥ 3) and a reasonable percentage of good guides, both methods do a reasonably good job in ranking the positive genes above the null genes, as indicated by the high AUC (Fig. 4 and Additional file 1: Figure S20). We suspect that this generalizes to other methods as well. On the other hand, we find that it is difficult to correctly identify all of the positive genes with a low number of sgRNAs. Even at 95% guide efficiency, 88% of true positive genes were identified at 3 sgRNAs per gene by CRISPhieRmix. Increasing the number of guides to 5 or 10 guides per gene increases the percentage of positive genes identified to 93% and 99%, respectively. Meanwhile, the ROC-AUC was equal to 0.97, 0.99, and 0.995 in these cases, indicating that the gene rankings were highly similar. The number of genes correctly identified by the Mann-Whitney test was lower (69%, 82%, and 97% at 3, 5, and 10 guides per gene, respectively).

We suspect that the low number of guides in current libraries prevent some truly interesting genes from being identified, either through varying sgRNA efficiency or through varying gene effect sizes. Genes with larger effect are easier to distinguish from null genes, and it takes more evidence to distinguish genes with small effects. This can only be done with more guides per gene. In our simulations, the effect size of the genes could be considered to be moderate but the resulting densities resemble densities of real screen (e.g., Additional file 1: Figure S17). The true genes will be easier to identify when genes have a larger effect. If researchers only want to investigate the few genes with the largest effects, then a smaller library will suffice, but we believe that 3 sgRNAs per gene severely limits the ability to investigate interesting biology.

## Discussion

We presented a new method of analyzing large-scale CRISPRi and CRISPRa screens that utilizes a hierarchical mixture model to allow for the possibility of varying guide efficiency and response. Though we presented our method primarily in terms of CRISPRi/a screens, we showed that it can be applied to CRISPRko screens if there is any possibility of varying guide efficiency. This problem is not nearly as pronounced in CRISPRko screens as it is in CRISPRi/a screens. Therefore, we expect that the improvement in such cases will only be small, but we showed that even in CRISPRko screens CRISPhieRmix is competitive with the current state of the art algorithms. We showed that the improvement in CRISPi/a screens is dramatic. The ability to model guides as a mixture distribution helps to distinguish genes that have varying guide efficiencies.

Our results indicate that a large number of guides and negative control guides may be unnecessary if researchers have plans to only study the top few genes, say if they decide beforehand to investigate the top 10 or 20 genes. The gene rankings can be estimated effectively without guides using a normal hierarchical mixture model or, in the case of no varying guide efficiency, a normal mixture model. This may help to save money, since the more guides requires higher sequencing depth, at the cost of decreased sensitivity. On the other hand, full discovery likely requires more guides than is currently standard. Five or fewer guides per gene may be inhibiting full investigation, as our results indicate that important genes can be missed in the case of varying guide efficiency.

Our method is specifically designed to rank genes and does not produce estimates of effect size, as MAGeCK MLE does. This is because of the identifiability issue in the simultaneous estimation of the gene and guide mixtures. To circumvent this issue, one could identify which genes are highly likely to be non-null and then estimate effect sizes only for these genes using a hierarchical mixture with *f*_1_ as a prior. Doing this, on the other hand, will result in an upwardly biased estimate of the percentage of guides that work, as genes that by random chance had few or no working guides in the screen will be excluded from this second-level analysis.

An interesting outcome of our simulations is that we sometimes observed low power for the Mann-Whitney test. This behavior of the Mann-Whitney test was surprising to us, as it is a workhorse method for non-parametric hypothesis testing and is typically suggested in small to moderate sample sizes [37], although in our case the sample size is both large (for the negative control guides) and small (for guides targeting a specific gene). Such a large difference in sample size is rarely encountered in the statistics literature and has not been investigated in depth. A subsequent literature search revealed recent discussions on issues with the Mann-Whitney test [38–40]. Though the two sample Mann-Whitney test is designed to test whether they arose from the same distribution, in reality, it can only detect divergences from Pr(*X*<*Y*)=0.5 [41]. Therefore, the Mann-Whitney test may have low power when the percentage of effective guides is below 50%. This is a possible explanation for the stark difference in performance between our CRISPRko and CRISPRi simulations.

We have made the CRISPhieRmix software available as an open source R package at https://github.com/timydaley/CRISPhieRmix. Detailed instructions and exampled are given in the manual, in the vignette section.

## Conclusion

Variable guide efficiency can create major issues in the analysis of CRISPRi/a screens, as well as CRISPRko screens. Accounting for this in the analysis greatly improves inferences and helps to identify genes that are likely to be related to the phenotype of interest but suffer from low guide efficiency. Our method, CRISPhieRmix, improves identification while effectively controlling the false discovery rate in both simulated and real data.

We used simulations to estimate how performance changes as a function of guide efficiency. These indicated that while it is easy to identify the genes with the highest effect, full discovery requires more than 3 guides per gene. This indicates that current studies may be using too few guides and researchers may by random chance be missing out on important biology.

## Methods

### Model

Let *g*_*i*_:{1,…,*N*}→{1,…,*G*} denote the mapping from sgRNAs to genes; *Z*_*g*_ be the latent indicator variable that gene *g* is interesting, with *Z*_*g*_∼Binomial(*p*); *Y*_*i*_ be the latent indicator variable that guide *i* works, with *Y*_*i*_=0 if $Z_{g_{i}} = 0$ (e.g., if the gene corresponding to guide *i* is null) and *Y*_*i*_∼Binomial(*q*) if $Z_{g_{i}} = 1$; and let *x*_*i*_ be the log2 fold change of guide *i*, typically computed by averaging the empirical log2 fold changes across replicates or by count analysis software such as DESeq2 [24] or edgeR [42]. Throughout the paper, we will use DESeq2-moderated log2 fold changes when raw counts are available, and provided log2 fold changes if the raw counts are not available.

The likelihood of the observed log2 fold changes is given by

1 $$\begin{array}{*{20}l} &\mathcal{L}(f_{0}, f_{1}, p, \pi | x_{i, j}, i = 1, \ldots, N, \, j = 1, \ldots, J) \\ & =\prod_{g = 1}^{G} (1 - p) \prod_{i: g_{i} = g} f_{0} (x_{i}) + p \prod_{i: g_{i} = g} \left((1 - q) f_{0} (x_{i}) + q f_{1} (x_{i})\right)\!.  \end{array} $$

The full likelihood, where the latent variables are assumed to be known, is given by

2 $$\begin{array}{*{20}l} &\mathcal{L} (f_{0}, f_{1}, p, \!\pi\! | x_{i, j}, Z_{g},\! Y_{i}; i\,=\, 1, \ldots, N,\! \, j \,=\, 1, \ldots, J, g \,=\, 1, \ldots, G) \\ &=\prod_{g = 1}^{G} \left(\prod_{i : g_{i} = g} f_{0}(x_{i}) \right)^{1 - Z_{g}} \left(\prod_{i : g_{i} = g} f_{0} (x_{i})^{1 - Y_{i}} f_{1}(x_{i})^{Y_{i}}\right)^{Z_{g}}.  \end{array} $$

### Identifiability

Note that the factorization (2) can be written as

3 $$  \prod_{i = 1}^{N} f_{0}^{\left(1 - Y_{i} Z_{g_{i}}\right)} f_{1}^{Y_{i} Z_{g_{i}}}.  $$

Therefore, if we were to use an EM algorithm in the straightforward manner, *p* and *q* are not simultaneously identifiable.

We can, however, estimate *τ*=*pq*, the probability that a randomly chosen guide arises from *f*_1_, with relative ease. Let $W_{i} = Y_{i} Z_{g_{i}}\phantom {\dot {i}\!}$. Then, the complete likelihood is given by

$$\prod_{i = 1}^{N} f_{0}^{(1 - W_{i})} f_{1}^{W_{i}} $$

and *W*_*i*_ is a Bernoulli (*τ*) random variable. We assume that *f*_1_ is the Normal (*μ*,*σ*^2^) density. Therefore, we can estimate *μ*, *σ*^2^, and *τ* with a simple EM algorithm, with the two cases discussed above: *f*_0_ fixed (when negative control guides are provided), or *f*_0_ a Normal$\left (\mu _{0}, \sigma ^{2}_{0}\right)$ density, and *μ*_0_ and $\sigma ^{2}_{0}$ estimated in the EM (when negative control guides are not provided).

Since we are only interested in determining the interesting genes, the primary quantities of interest are the posterior probabilities that each gene is interesting. In this case, *q*, the mixing parameter on guides for the interesting genes, is a nuisance parameter. As is standard, we can eliminate *q* by marginalization. Let $\hat {\tau }$ denote the estimated value of *τ*=*pq*. The gene-level posterior probability for gene *g* is then given by

4 $$  {}\begin{aligned} \int_{0}^{1} \frac{(\hat{\tau} / q) \prod_{i: g_{i} = g} \big(q f_{1} (x_{i}) + (1 - q) f_{0} (x_{i}) \big)} {(\hat{\tau} / q) \prod_{i: g_{i} = g} \big(q f_{1} (x_{i}) + (1 - q) f_{0} (x_{i}) \big) + (1 - (\hat{\tau} / q)) \prod_{i: g_{i} = g} f_{0} (x_{i})} d \psi (q), \end{aligned}  $$

where *ψ*(*q*) is the prior distribution of *q*. We typically set *ψ*(*q*) to be the uniform distribution in case of no prior knowledge of *q*. It should be noted that *ψ* cannot have support less than *pq*, as this would result in *p*>1.

To speed up calculation of the quantity (4), we use Gaussian quadrature [43] when possible. For example, when *ψ*(*q*) is a uniform distribution, the weights and points can be calculated from transformed Legendre polynomials, which are precomputed to speed up calculation.

Above we assumed that *f*_1_ is a unimodal distribution, as is the case when one is interested in a unidirectional phenotype (e.g., essential genes). If one is interested in a bidirectional phenotype (e.g., drug resistance and susceptibility), then extending the framework above to allow for *f*_1_ to be a bimodal distribution is straightforward and is available as an option in the CRISPhieRmix software.

### The local false discovery rate

The local false discovery rate (fdr) for gene *g* is defined as [17, 18]

$$\text{fdr}_{g} = \Pr(\text{gene}\ g\ \text{is uninteresting} | x_{i} : g_{i} = g). $$

Note that in our mixture model, this is also equal to the posterior probability that gene *g* is null. In other words, fdr_*g*_ is equal to one minus the posterior probability of being interesting, which is given in Eq. (4).

The local fdr tends to be less restrictive than the global FDR, which is defined as the expected fraction of incorrectly identified genes at a given threshold. We can see this by considering a univariate *x* and a one-sided test. The local fdr is equal to Pr(uninteresting|*X*=*x*) while the global FDR is equal to Pr(uninteresting|*X*≥*x*), so that one can think of the local fdr as akin to the probability density function (pdf) while the global FDR is akin to the cumulative distribution function (cdf). In our multivariate setting, this relationship is not so simple as we must identify the level sets of the local fdr to integrate over the boundary. This will be extremely difficult as the level sets are not convex. In two dimensions, they will look similar to a four-pointed star, and in higher dimensions, they are likely even more complicated.

Instead we can use the empirical distribution of the local fdr to estimate the global FDR. If *s*_*g*_,*g*=1,…,*G* are the gene scores and *s*_(1)_,…,*s*_(*G*)_ are the ordered gene scores, then the genes called at a global FDR of *π* satisfy

$$s_{(1)}, \ldots, s_{(K)}: \frac{1}{K} \sum\limits_{k = 1}^{K} s_{(k)} \leq \pi. $$

As a sanity check, consider the extreme example of averaging across all genes. Then, the global FDR is estimated as the proportion of genes that are null, exactly what we would expect.

### The skew-*t* distribution

A real valued random variable *X* follows a skew-*t* distribution with parameters *ξ*,*ω*,*α*,*ν* (written as $X \sim \mathcal {ST} (\xi, \omega, \alpha, \nu)$) if it has density

$$f(y; \xi, \omega, \alpha, \nu)\,=\,\! \frac{2}{\omega} t_{\nu}\! \left(\!\frac{y - \xi}{\omega}\!\right) \!\!T_{\nu + 1} \!\!\left(\!\alpha \frac{y - \xi}{\omega} \!\!\!\sqrt{\frac{\nu + 1}{\nu + \left(\frac{y - \xi}{\omega}\right)^{2}}} \right)\!, $$

where *t*_*ν*_ and *T*_*ν*_ respectively denote the pdf and cdf of a *t* distribution with *ν* degrees of freedom [44].

The R package sn [45] provides procedures for obtaining maximum likelihood estimates and density estimates.

## Additional file

Additional file 1 **Figure S1–S20.** Supplementary Information. (PDF 6653 kb)
