# Supplementary material for: CRISPhieRmix: a hierarchical mixture model for CRISPR pooled screens
Source: Genome Biol. 2018 Oct 8;19:159. doi: 10.1186/s13059-018-1538-6 (PMC6176515; doi:10.1186/s13059-018-1538-6)
Supplement: Supplementary file 1 — Figure S1–S20. Supplementary Information. (PDF 6653 kb) [file 13059_2018_1538_MOESM1_ESM.pdf]

A hierarchical mixture model for CRISPR pooled screens

Timothy Daley[1,2], Xueqiu Lin[1], Yanxia Liu[1], Zhixiang Lin[2], Lei S. Qi[1,3,4,6,*], and Wing Hung Wong[2,5,*]

[1]Department of Bioengineering, Stanford University

[2]Department of Statistics, Stanford University

[3]Chemical and Systems Biology, Stanford University

[4]ChEM-H Institute, Stanford University

[5]Department of Biomedical Data Science, Stanford University

[6]To whom correspondence should be addressed to: tdaley@stanford.edu, stanley.qi@stanford.edu, and whwong@stanford.edu

[*]These authors jointly supervised this work

Supplementary Figures

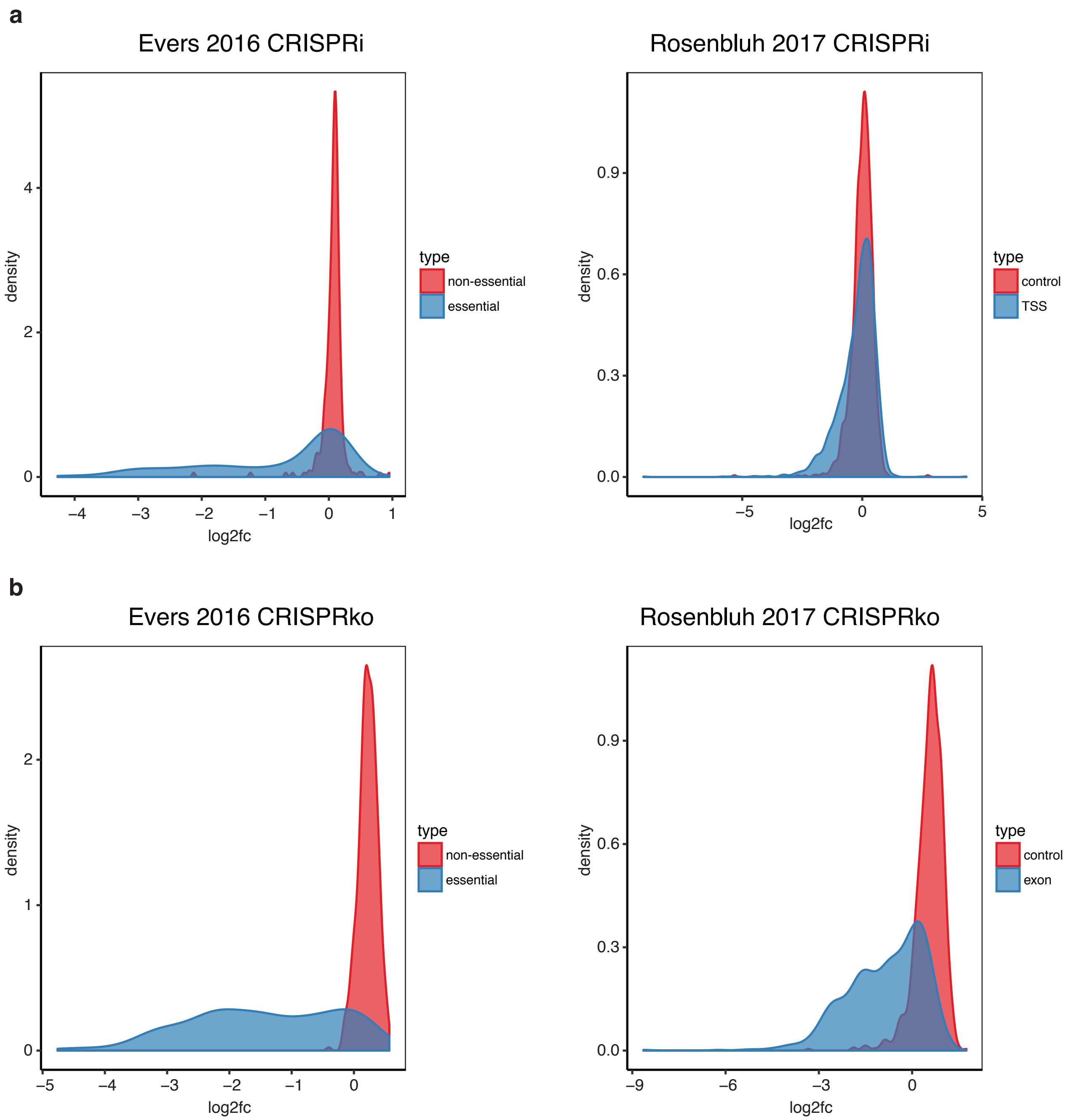

Figure S1: DESeq2 normalized log2 fold changes for known essential genes, non-essential genes, and negative control guides in Evers *et al.* 2016 and Rosenbluh *et al.* 2017 in **a** CRISPRko and **b** CRISPRi screens.

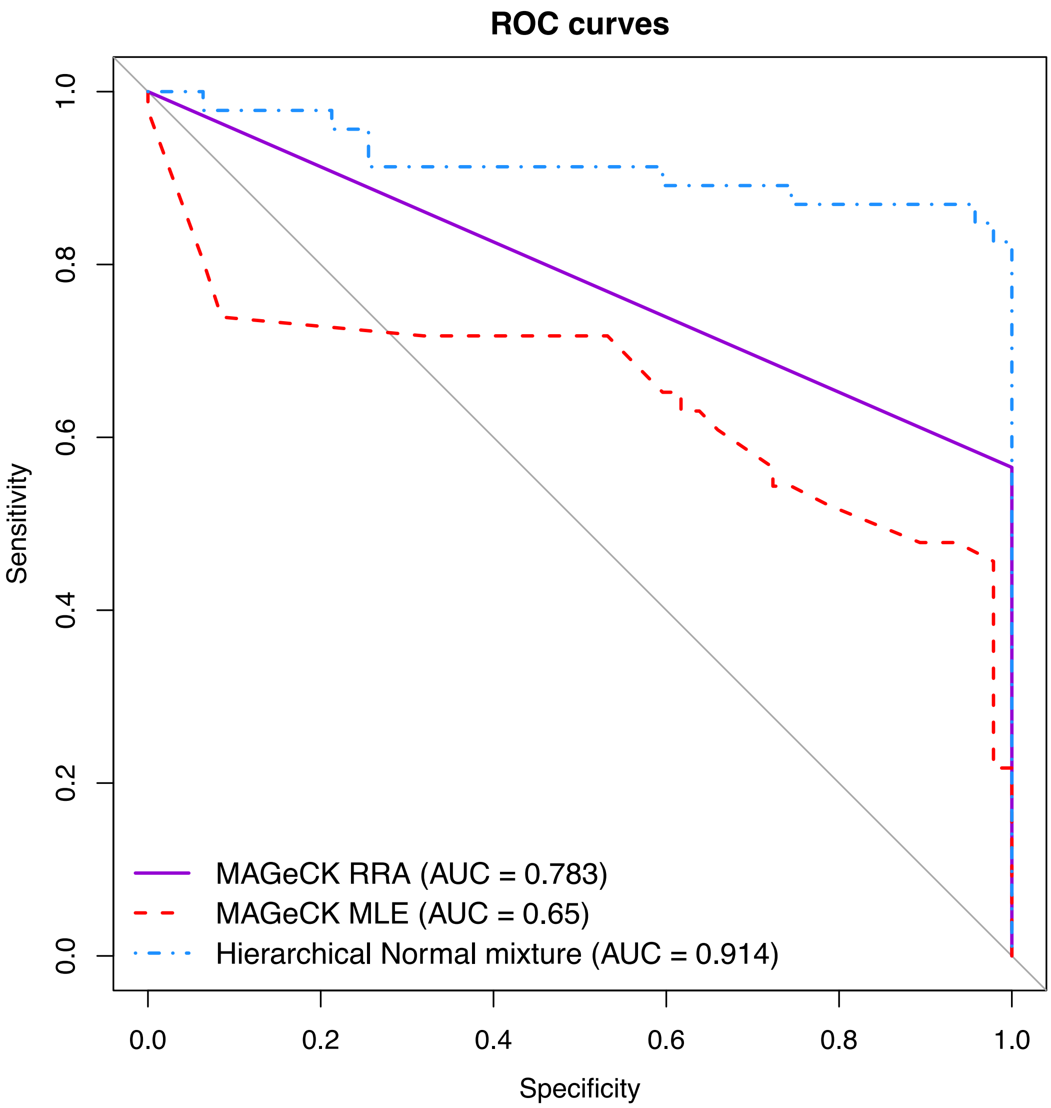

Figure S2: ROC curves for our hierarchical normal mixture, MAGeCK RRA, and MAGeCK MLE on data from Evers *et al.* 2016.

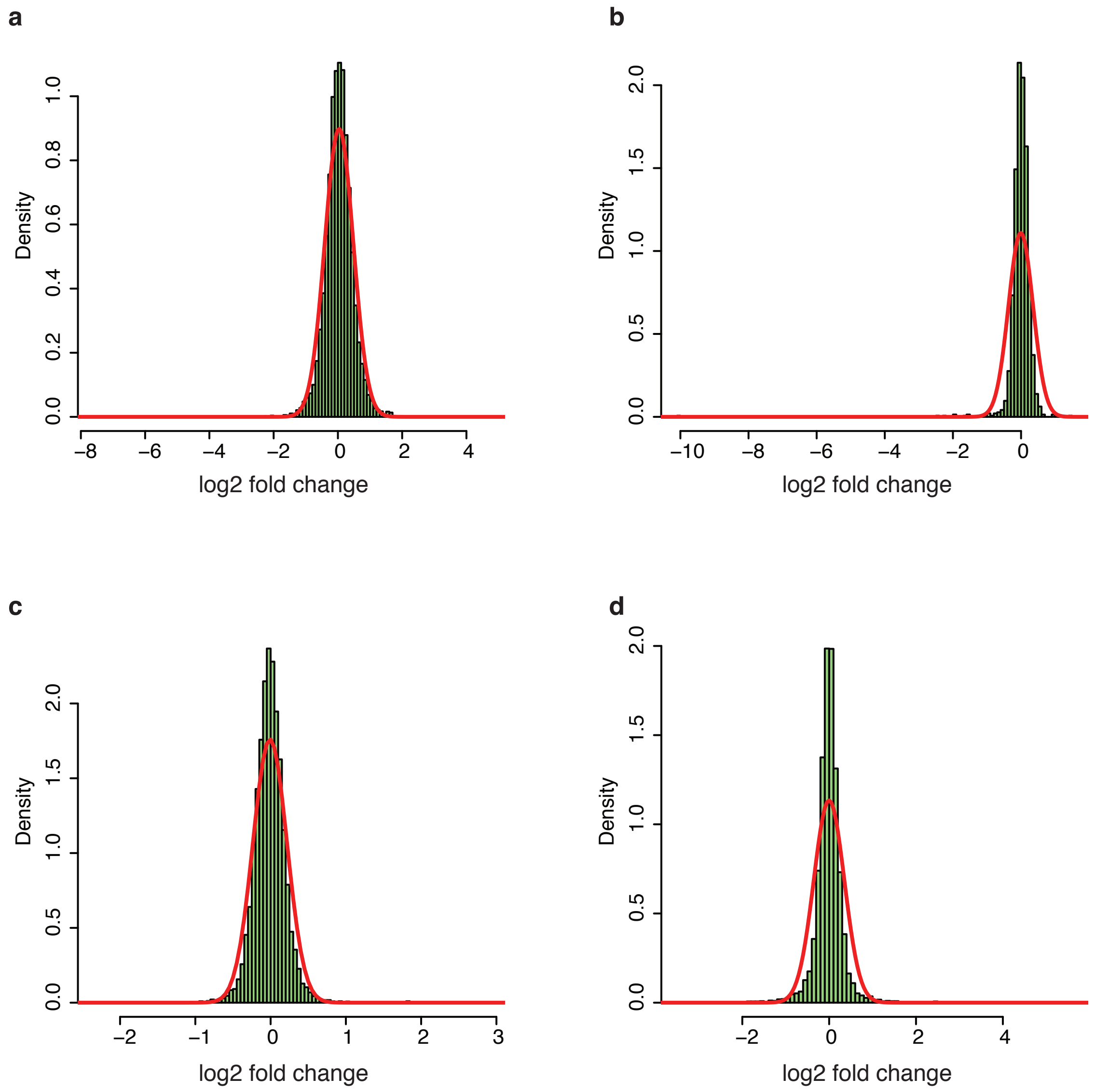

Figure S3: Normal fit to DESeq2 normalized log2 fold change of the negative control guides of **a** a CRISPRi screen for ricin phenotypes (Gilbert *et al.* 2014); **b** a CRISPRi screen for functional lncRNAs in HeLa cells (Liu *et al.* 2017); and **c** a CRISPRi screen and **d** a CRISPRa screen for rigosertib phenotypes (Jost *et al.* 2017).

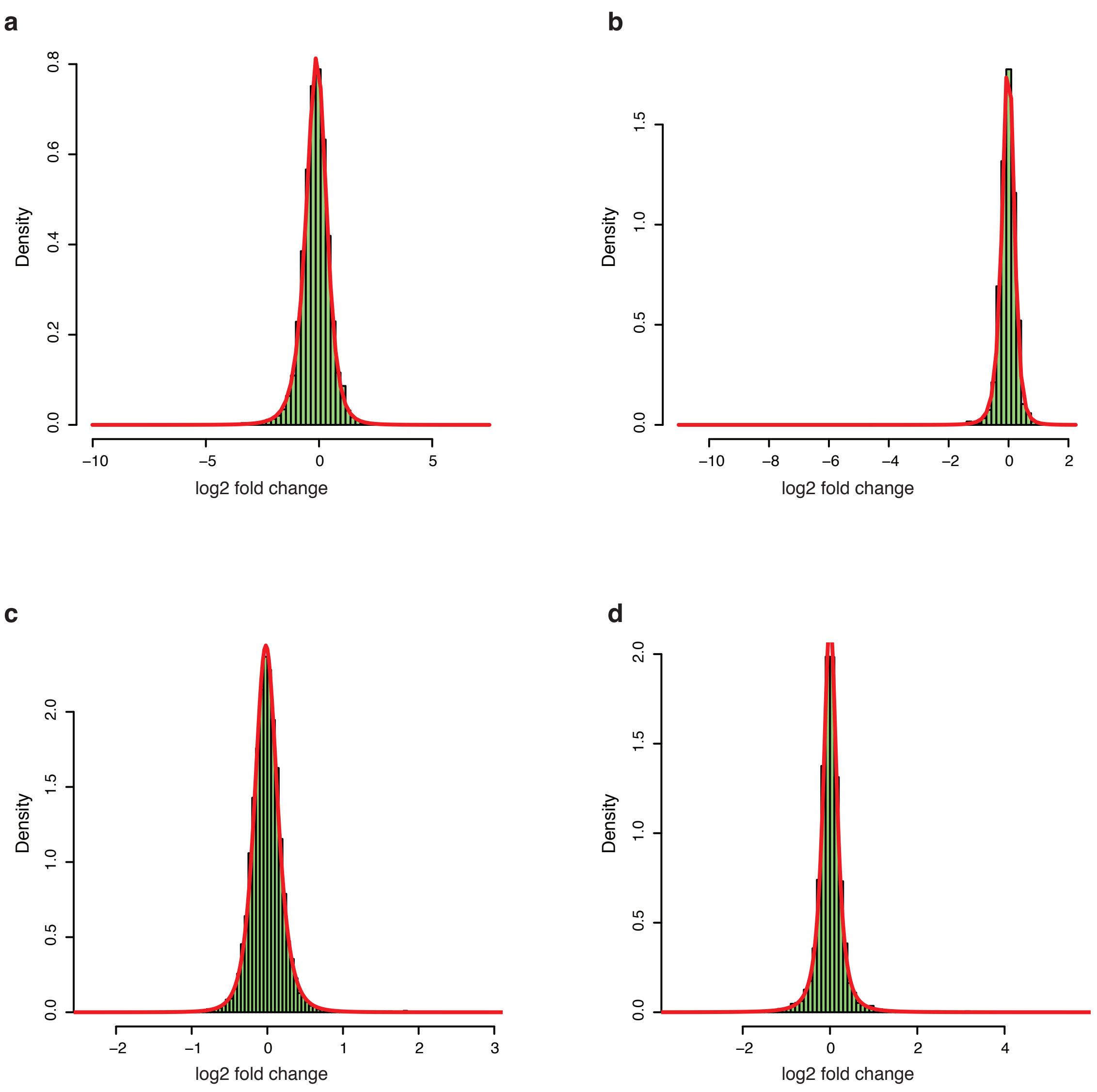

Figure S4: Skew-t fit to DESeq2 normalized log2 fold change of the negative control guides of **a** a CRISPRi screen for ricin phenotypes (Gilbert *et al.* 2014); **b** a CRISPRi screen for functional lncRNAs in HeLa cells (Liu *et al.* 2017); and **c** a CRISPRi screen and **d** a CRISPRa screen for rigosertib phenotypes (Jost *et al.* 2017).

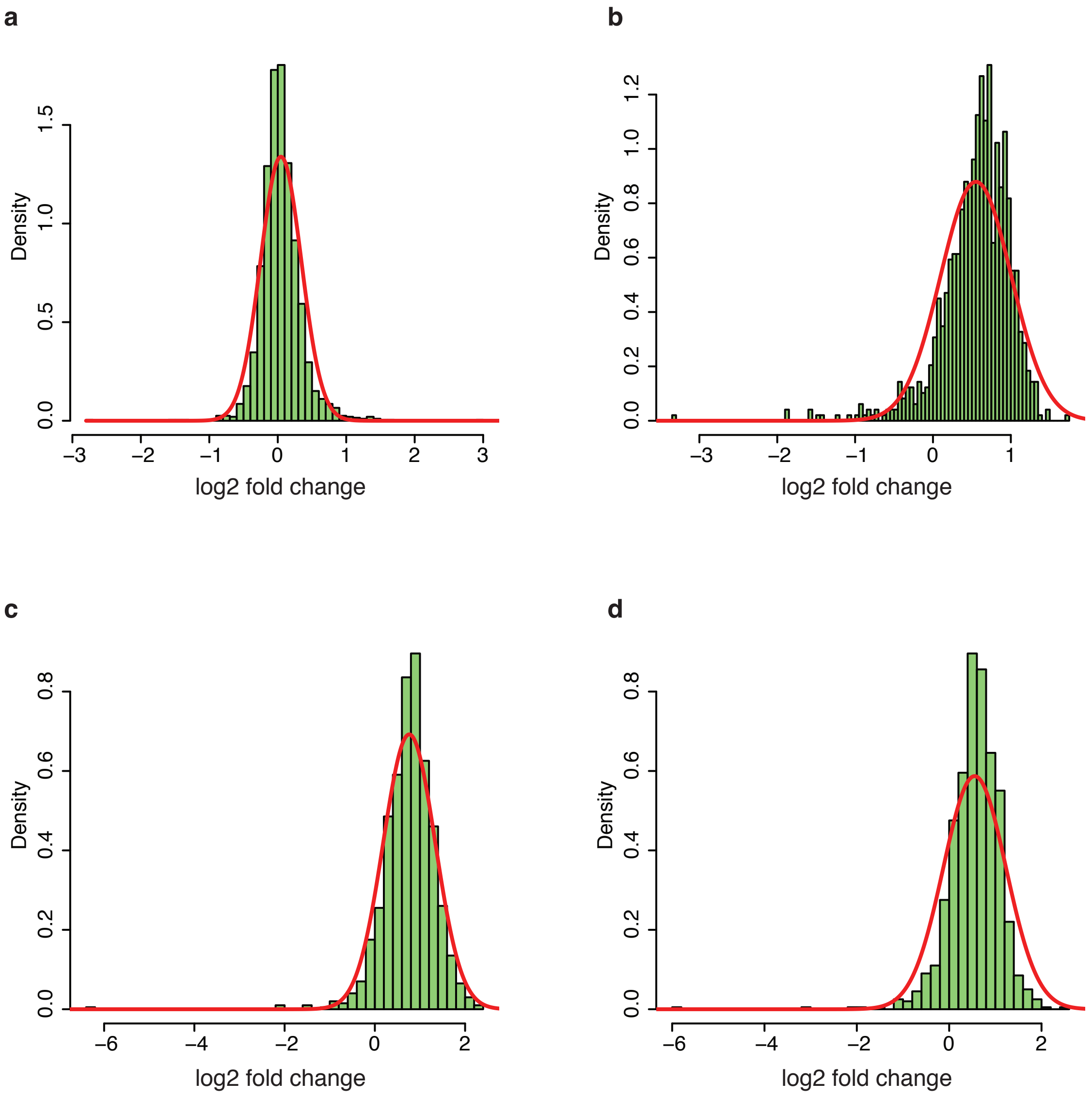

Figure S5: Normal fit to DESeq2 normalized log2 fold change of the negative control guides of CRISPRko screens of **a** a genome-wide screen for essential genes in K562 cells from Morgens *et al.* 2016, **b** a targeted loss of function screen in HT29 cells from Rosenbluh *et al.* 2017, **c** a genome-wide screen for essential genes in CADOES1_BONE cell line from Project Achilles (Aguirre *et al.* 2016), **d** a genome-wide screen for essential genes in EWS502_BONE cell line from Project Achilles.

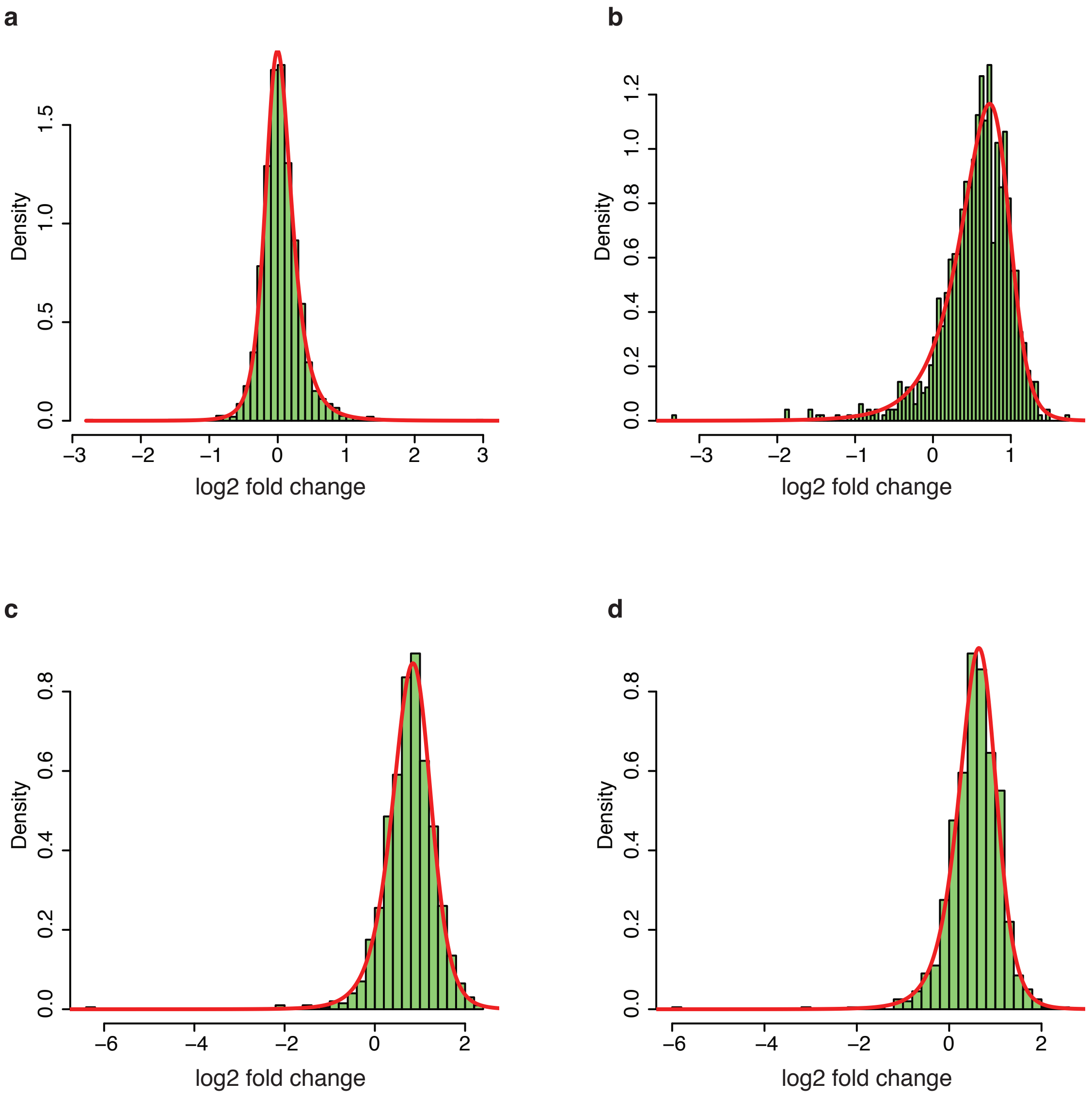

Figure S6: Skew-t fit to DESeq2 normalized log2 fold change of the negative control guides of CRISPRko screens of **a** a genome-wide screen for essential genes in K562 cells from Morgens *et al.* 2016, **b** a targeted loss of function screen in HT29 cells fromRosenbluh *et al.* 2017, **c** a genome-wide screen for essential genes in CADOES1_BONE cell line from Project Achilles (Aguirre *et al.* 2016), **d** a genome-wide screen for essential genes in EWS502_BONE cell line from Project Achilles.

Experiment	normal BIC	skew-t BIC
Morgens (2016)	842.62	**381.46**
Rosenbluh (2017) HT29_Cas9	1242.65	**998.49**
Project Achilles A375_SKIN	1235.27	**1145.50**
Project Achilles A673_BONE	2069.89	**2023.40**
Project Achilles BXPC3_PANCREAS	1695.90	**1648.41**
Project Achilles CADOES1_BONE	1747.04	**1566.99**
Project Achilles CAL120_BREAST	1425.47	**1401.62**
Project Achilles COLO741_SKIN	2075.87	**1862.86**
Project Achilles CORL105_LUNG	1560.34	**1513.67**
Project Achilles EW8_BONE	1627.58	**1572.22**
Project Achilles EWS502_BONE	1699.20	**1503.36**
Project Achilles G402_SOFT_TISSUE	1291.76	**1229.93**
Project Achilles HCC44_LUNG	1257.20	**1230.36**
Project Achilles HS294T_SKIN	1664.59	**1662.08**
Project Achilles HT29_LARGE_INTESTINE	1499.15	**1447.22**
Project Achilles K562_HAEMATOPOIETIC_AND_LYMPHOID_TISSUE	1144.83	**1129.79**
Project Achilles L33_PANCREAS	2074.34	**2062.36**
Project Achilles LNCAPCLONEFGC_PROSTATE	1594.97	**1439.68**
Project Achilles MEWO_SKIN	1961.62	**1918.46**
Project Achilles MHHES1_BONE	1621.00	**1619.15**
Project Achilles NCIH1373_LUNG	1294.75	**1213.40**
Project Achilles NCIH2009_LUNG	1555.25	**1435.12**
Project Achilles PANC0327_PANCREAS	**1222.87**	1226.74
Project Achilles PANC0813_PANCREAS	1500.67	**1464.10**
Project Achilles PANC1_PANCREAS	1301.01	**1252.16**
Project Achilles PATU8902_PANCREAS	1031.82	**920.00**
Project Achilles PATU8988T_PANCREAS	1416.30	**1402.10**
Project Achilles PC3_PROSTATE	1843.32	**1807.19**
Project Achilles RDES_BONE	1621.64	**1517.61**
Project Achilles SKES1_BONE	1896.23	**1861.25**
Project Achilles SU8686_PANCREAS	1517.81	**1463.64**
Project Achilles T47D_BREAST	1809.95	**1774.85**
Project Achilles TC32_BONE	1673.80	**1612.58**
Project Achilles TC71_BONE	2088.95	**2055.35**
Project Achilles TOV112D_OVARY	1633.98	**1607.19**

Table S1: Bayesian Information Criteria (BIC) of the normal model and the skew-t fit for the negative control guides of the indicated CRISPRko experiments. A smaller BIC is preferred and is in bold for each experiment.

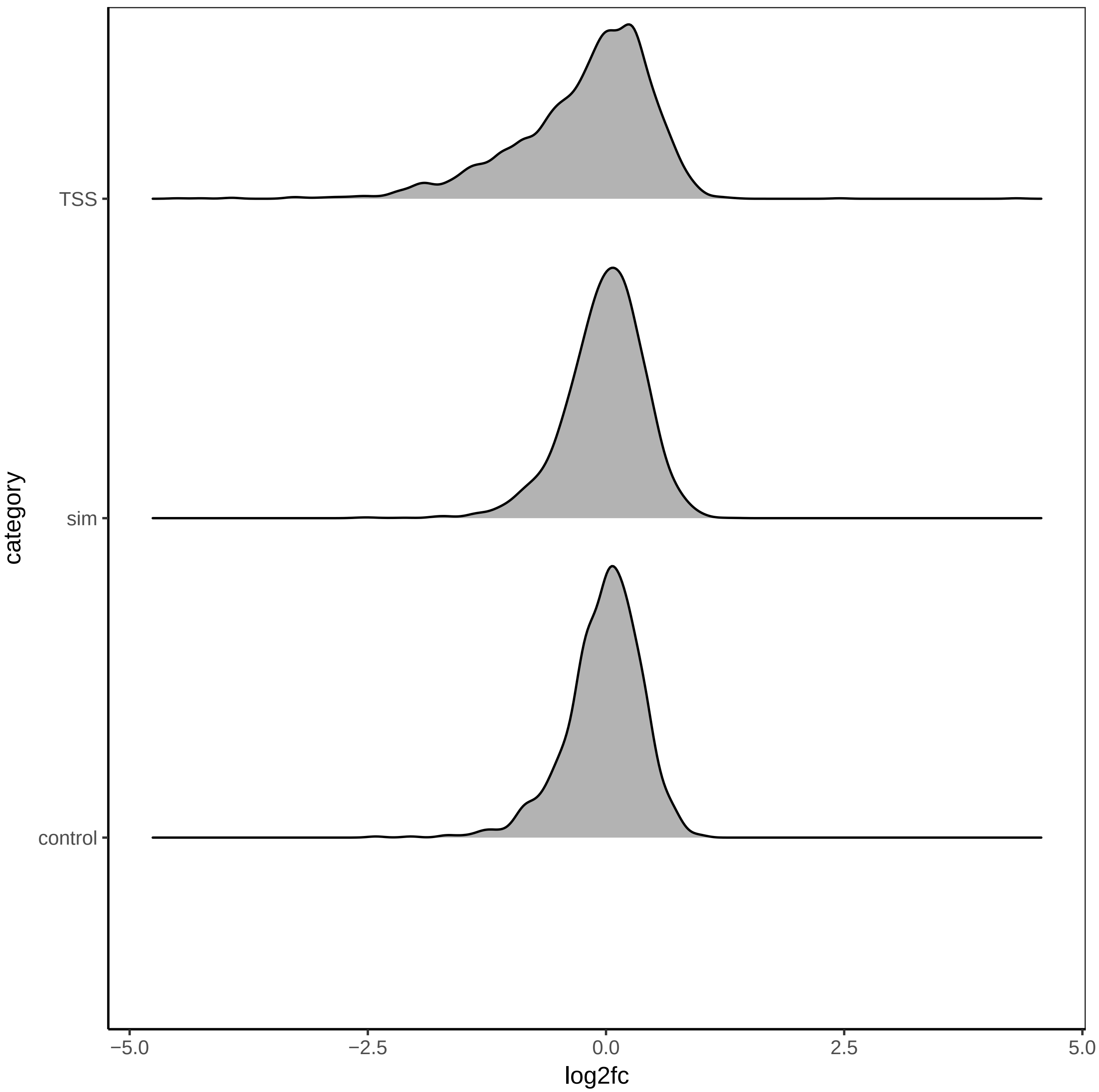

Figure S7: DESeq2 normalized log2 fold changes for positive essential genes, simulated negative genes, and negative control guides.

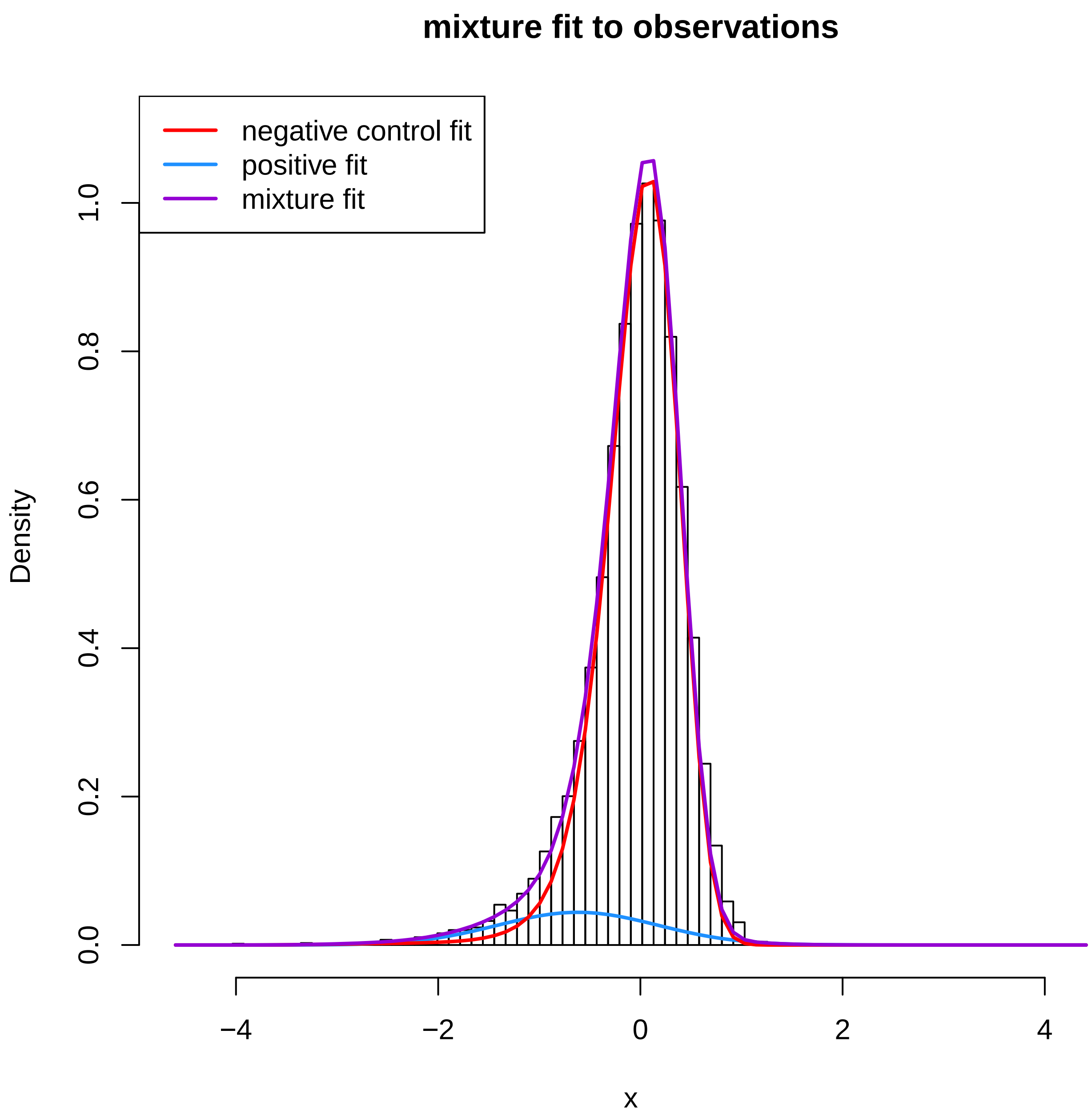

Figure S8: DESeq2 normalized log2 fold changes for the non-control guides, along with the mixture distribution fits.

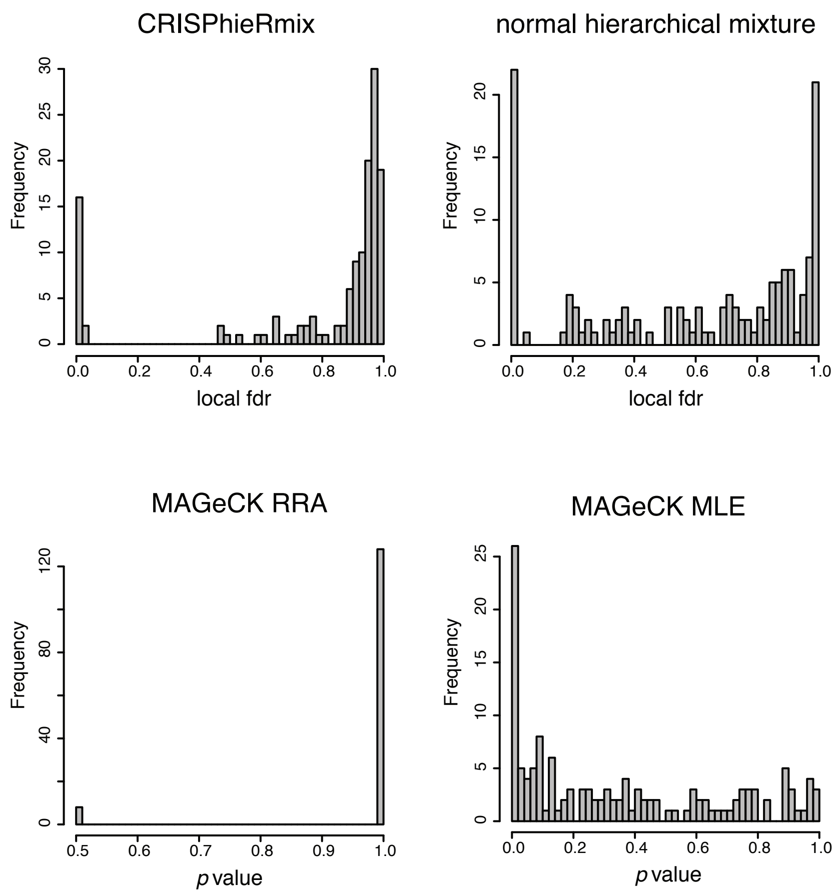

Figure S9: The estimated local fdr values estimated by CRISPhieRmix and the p-values obtained from MAGeCK RRA and MAGeCK MLE.

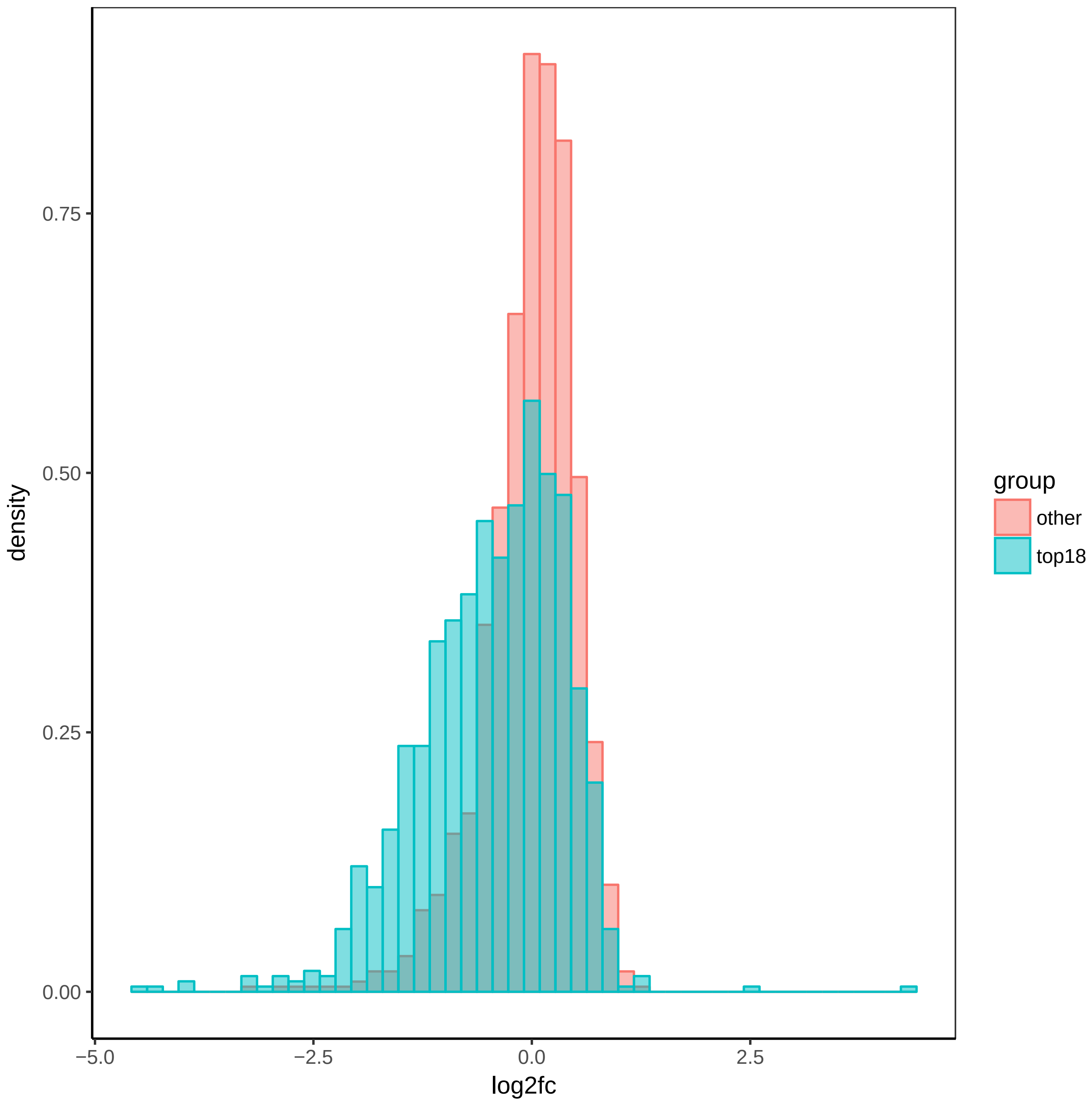

Figure S10: DESeq2 normalized log2 fold changes for the top 18 genes, clearly identified by CRISPhieRmix, and the other essential genes. A clear separation is observed between the two groups.

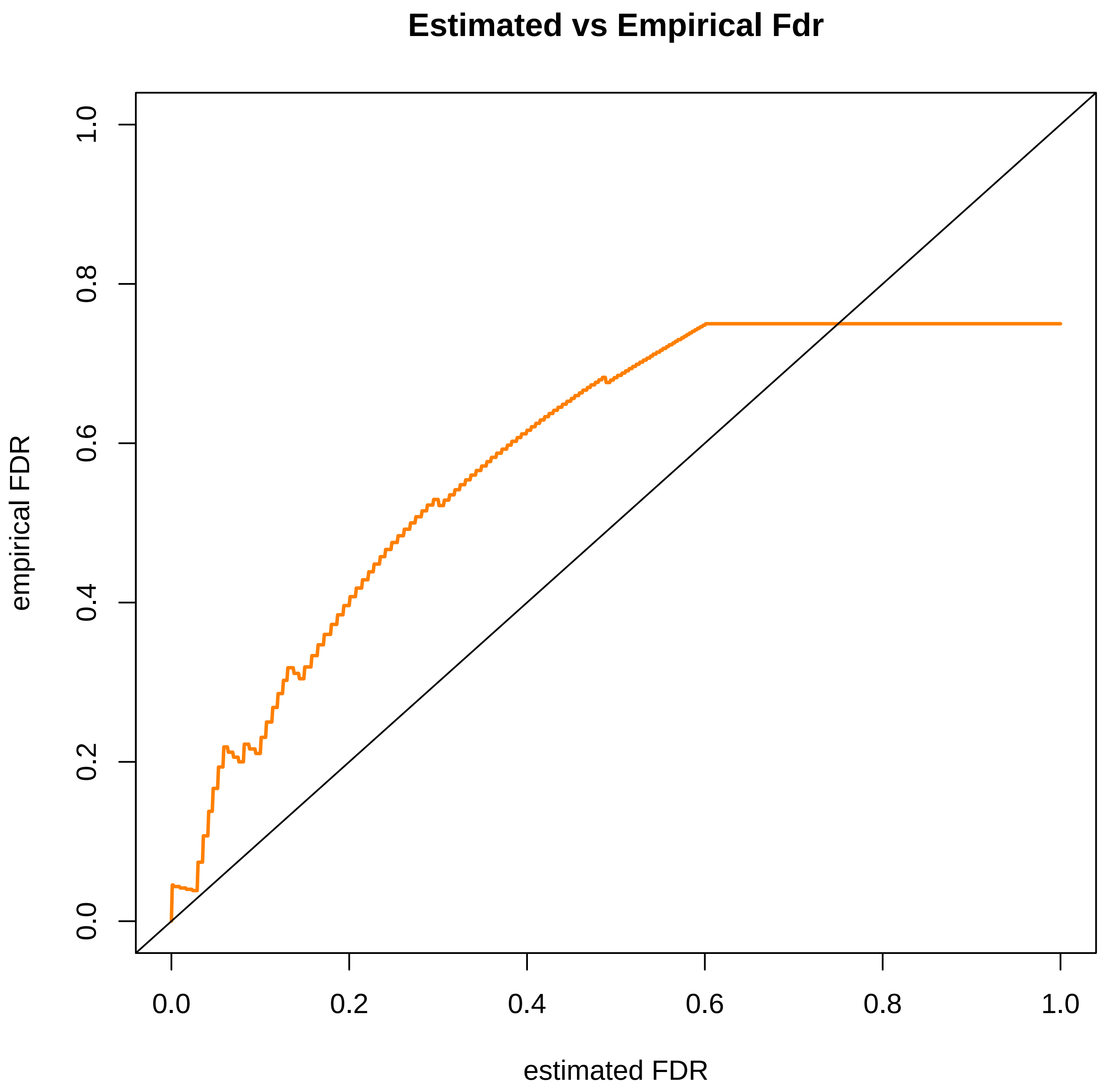

Figure S11: Estimated FDR plotted against the empirical FDR for the normal mixture model when applied to the Rosenbluh CRISPRi simulated data.

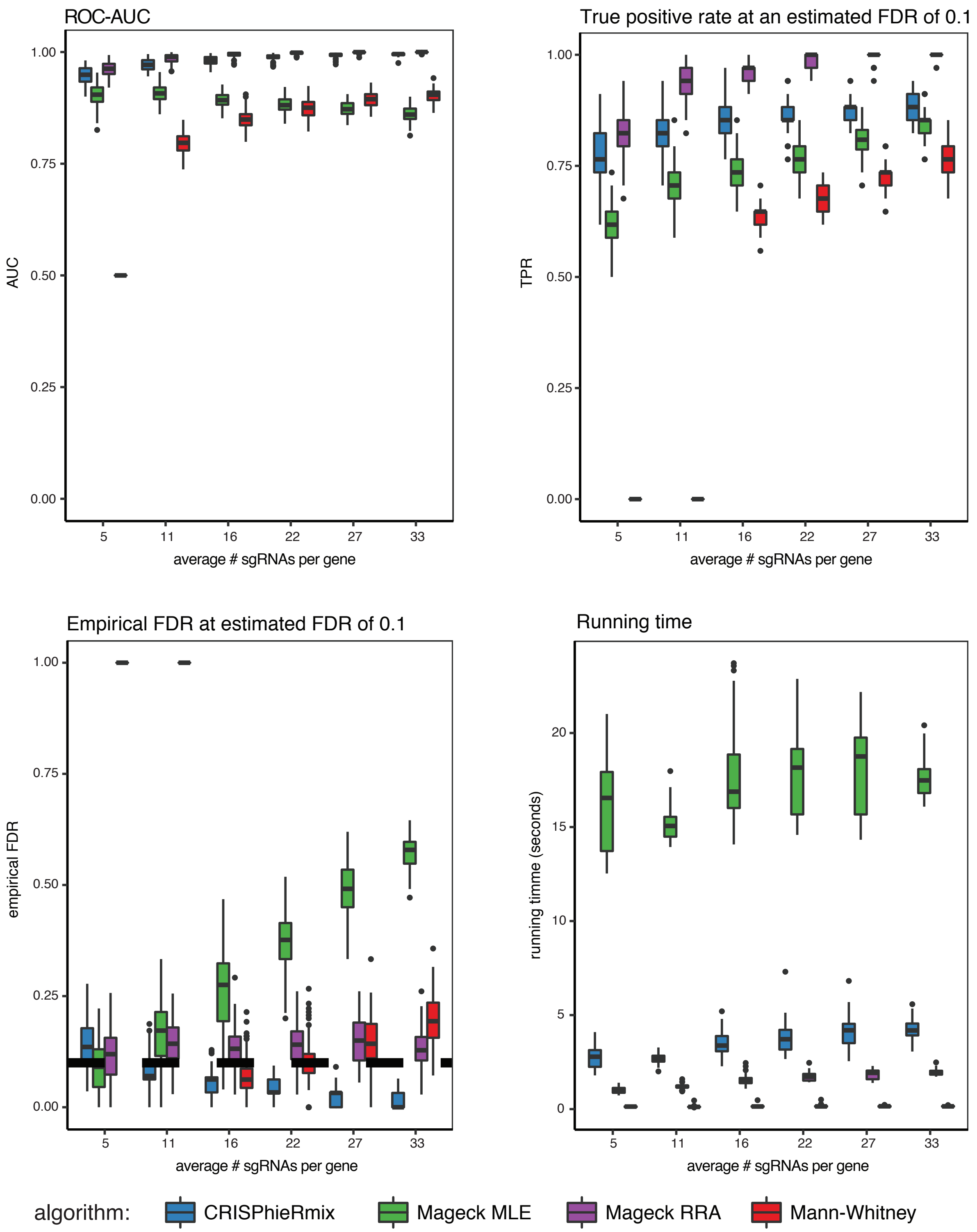

Figure S12: A comparison of algorithms as a function of sgRNAs per gene in the simulated CRISPRko screen. The area under the receiver operator curves (AUC), true positive rate (TPR) at a threshold of 0.1 global false discovery rate (FDR), empirical FDR at an estimated FDR of 0.1, and running times of all algorithms are shown. The dashed line indicates an FDR of 0.1, or perfect control of the FDR.

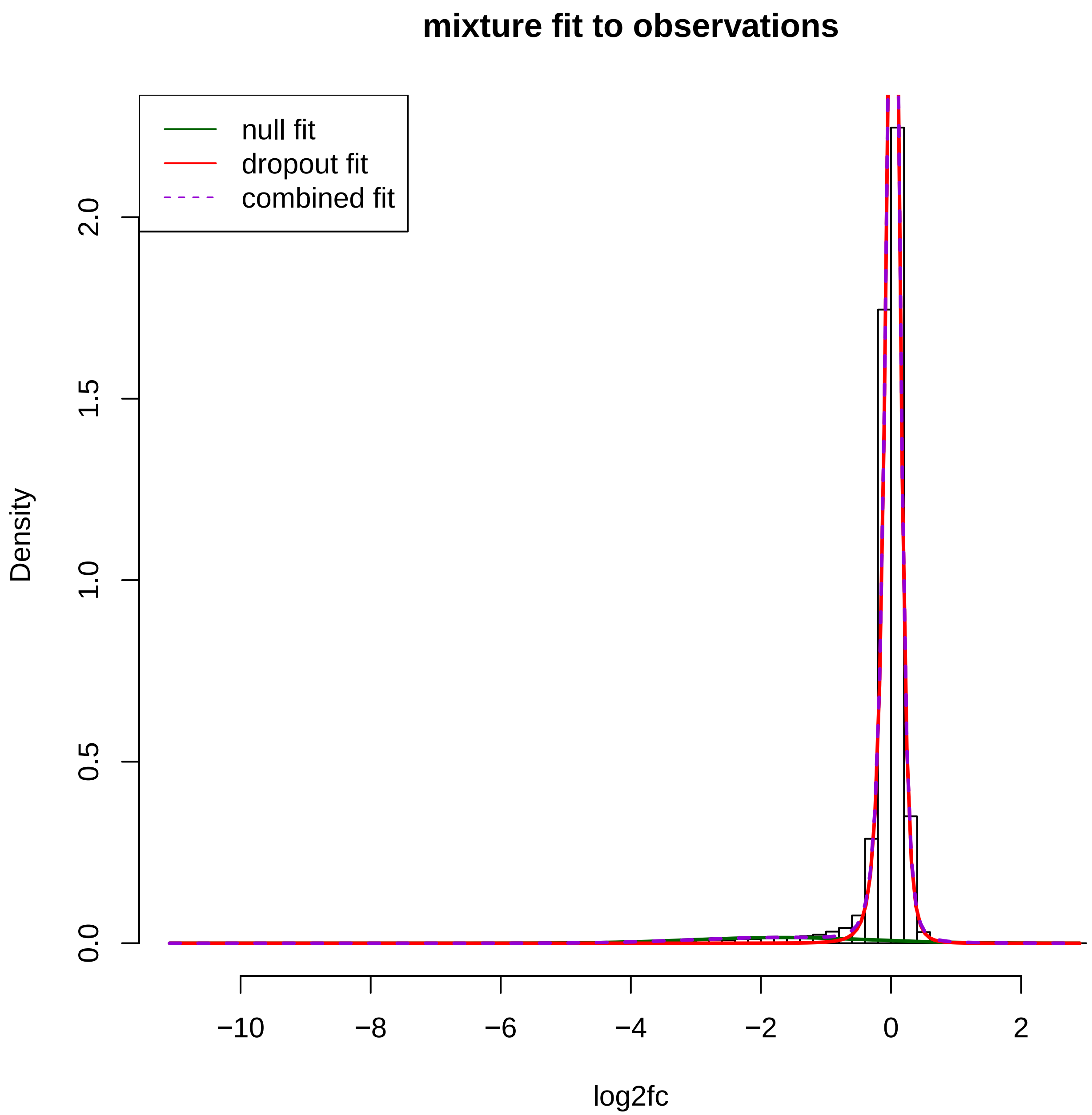

Figure S13: CRISPhieRmix fit to the $\log_2$ fold changes of the untreated population relative to the initial population.

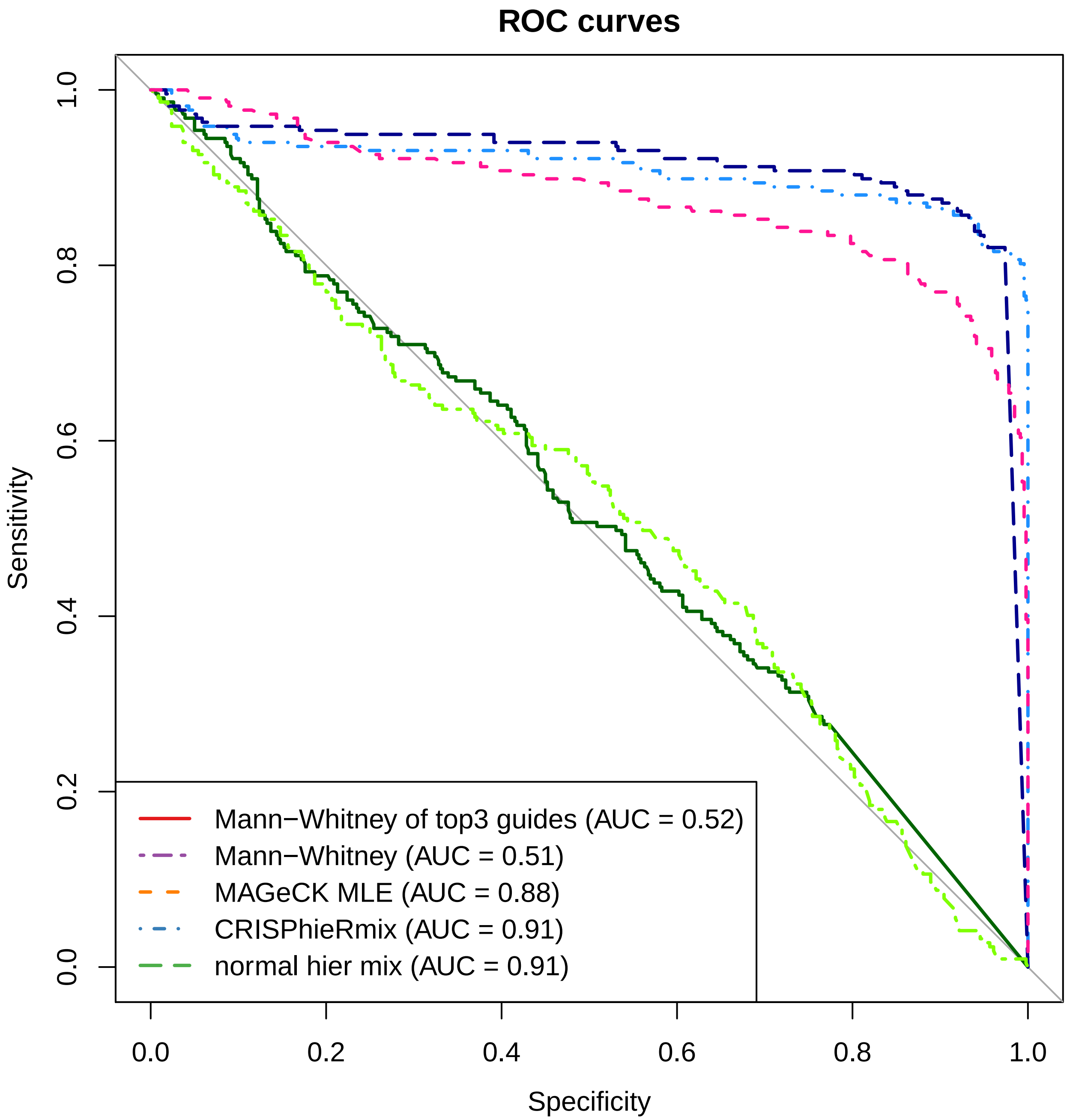

Figure S14: ROC curves for known essential and non-essential genes for a CRISPRi screen from Gilbert *et al.* 2014.

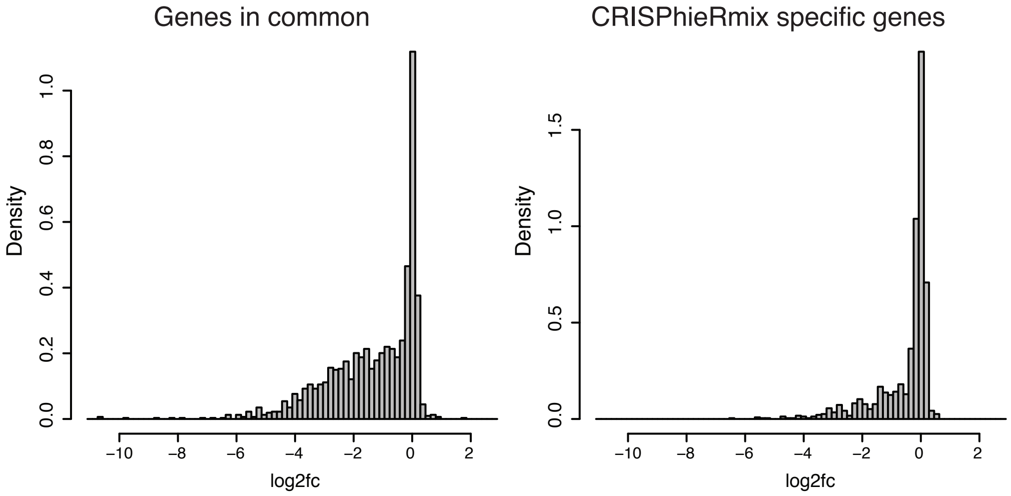

Figure S15: Histogram of $\log_2$ fold changes of guides corresponding to the 105 essential genes called by both MAGeCK MLE and CRISPhieRmix (left) and the 67 essential genes called only by CRISPhieRmix.

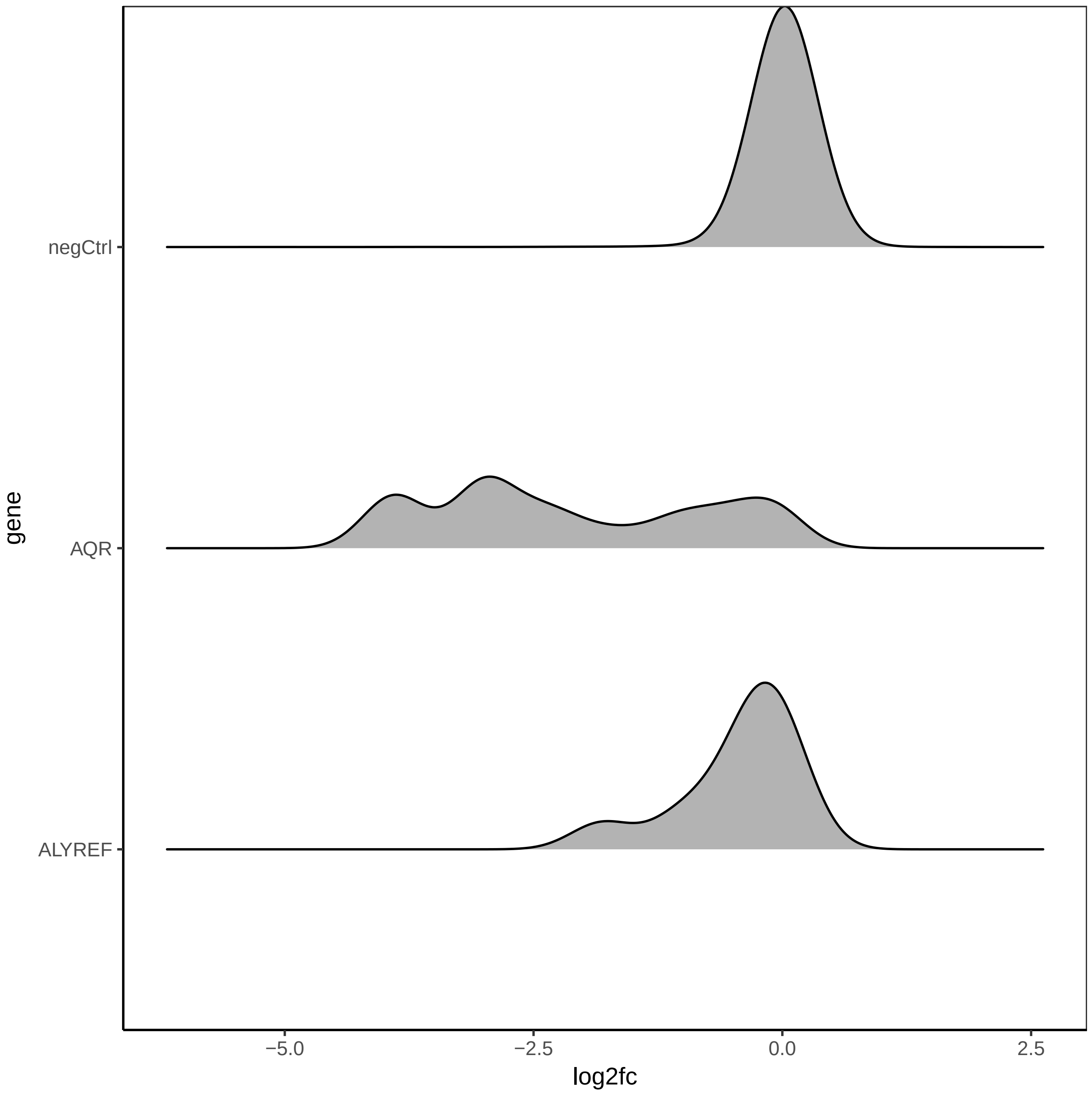

Figure S16: Gaussian kernel smoothed densities of negative control guides and two example genes. AQR was identified by both MAGeCK MLE and CRISPhieRmix, while ALYREF was identified by only CRISPhieRmix. Both genes are first in alphabetical order in their respective classes.

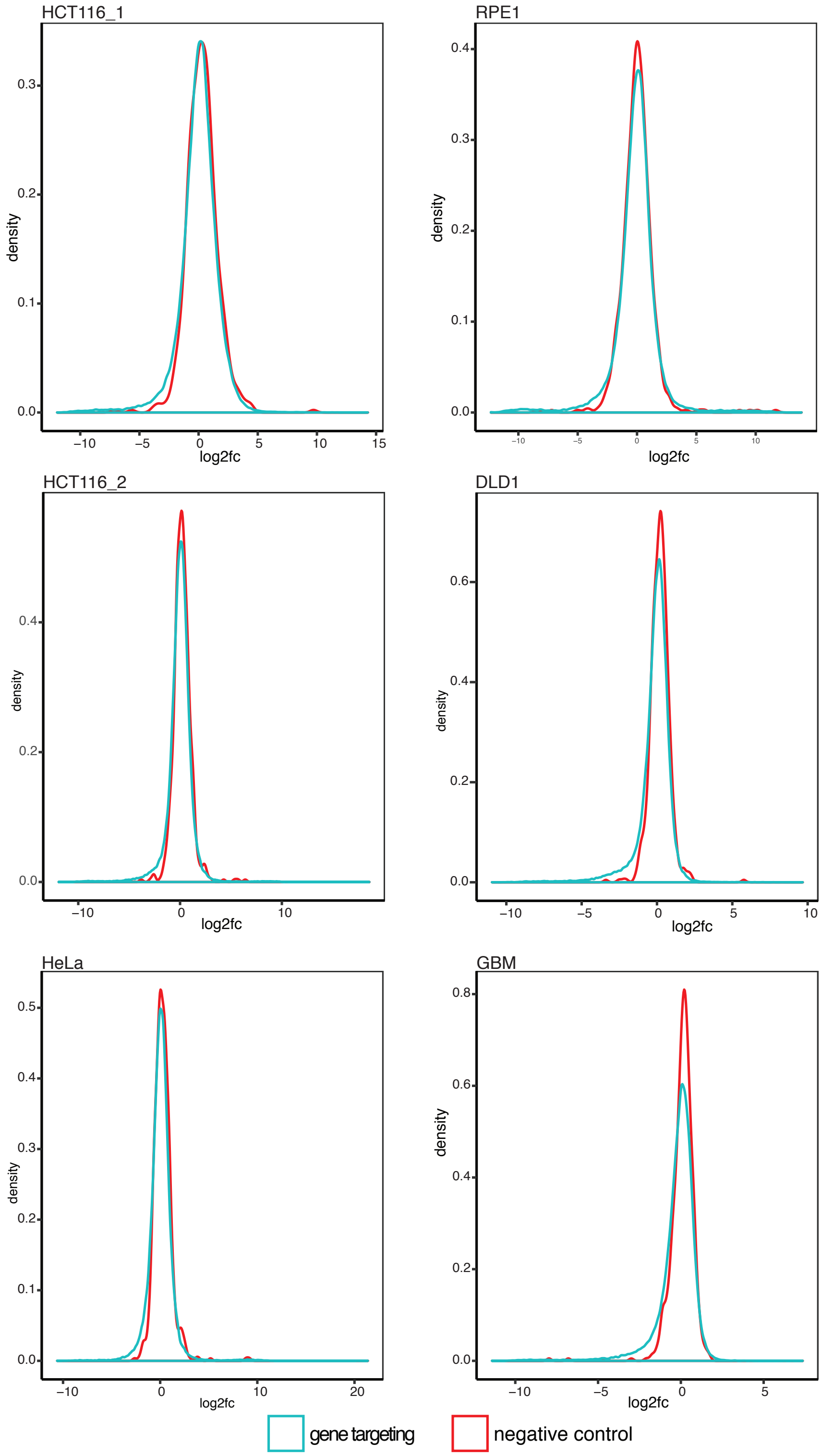

Figure S17: Density plots of ESeq2 calculated log2 fold changes for negative control guides and gene targeting guides.

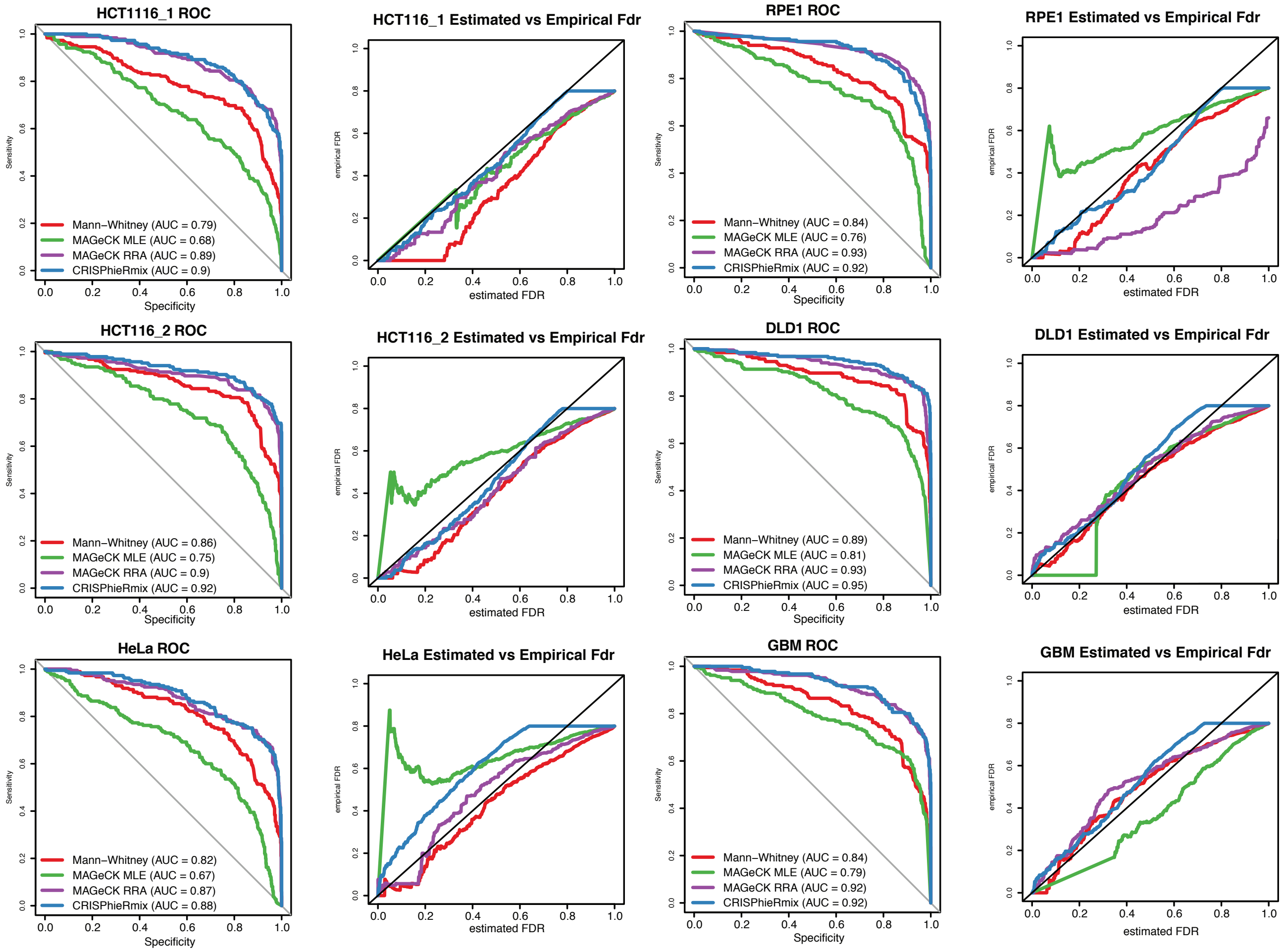

Figure S18: Evaluation of CRISPhieRmix, MAGeCK RRA, MAGeCK MLE, and the Mann-Whitney test on the 6 base library experiments of Hart *et al.* 2015 using the reference sets of Hart *et al.* 2014.

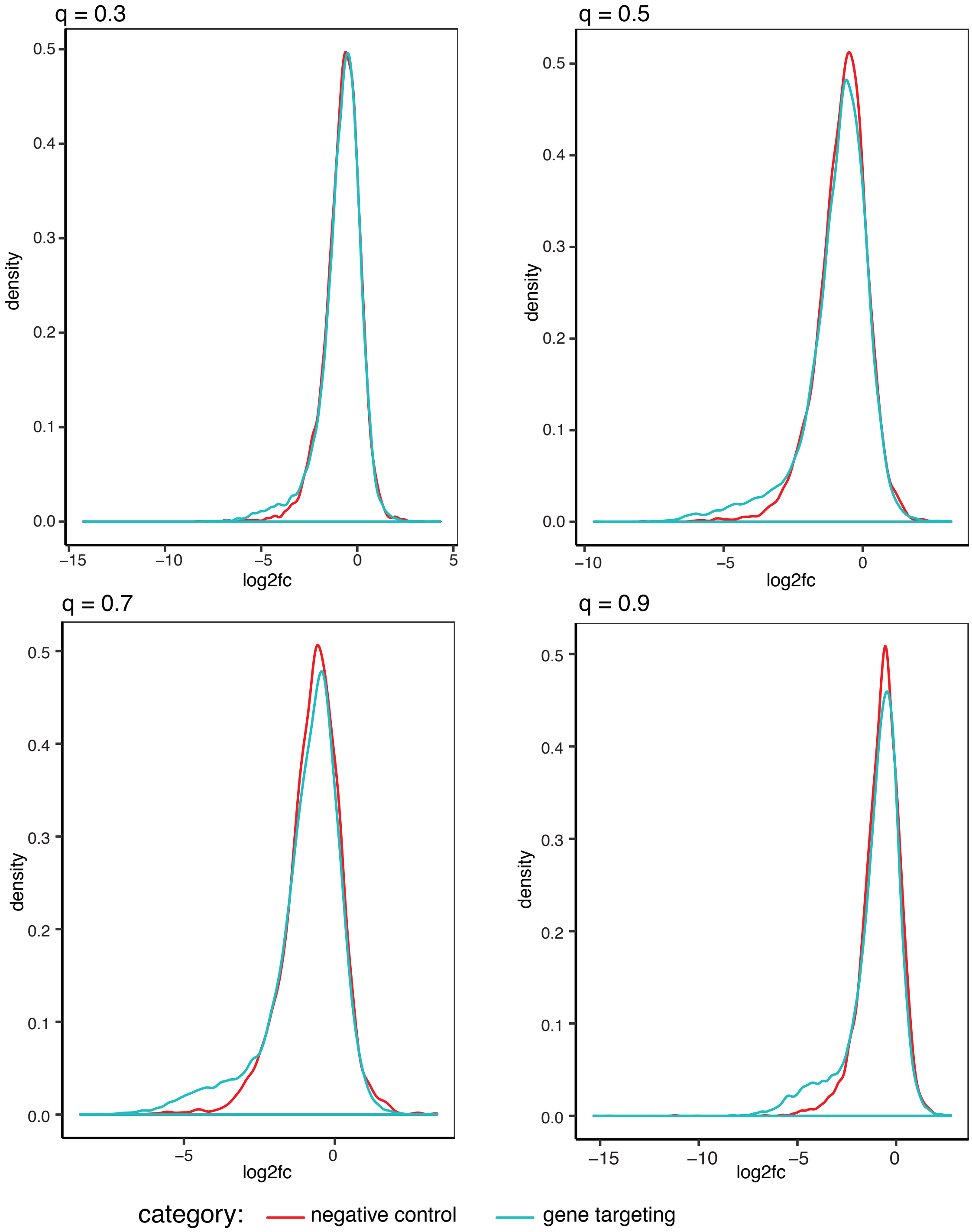

Figure S19: Example empirical densities of $\log_2$ fold changes for simulated gene targeting guides and negative control guides with varying guide efficiencies, indicated above each plot. Details on simulation are available in the main text.

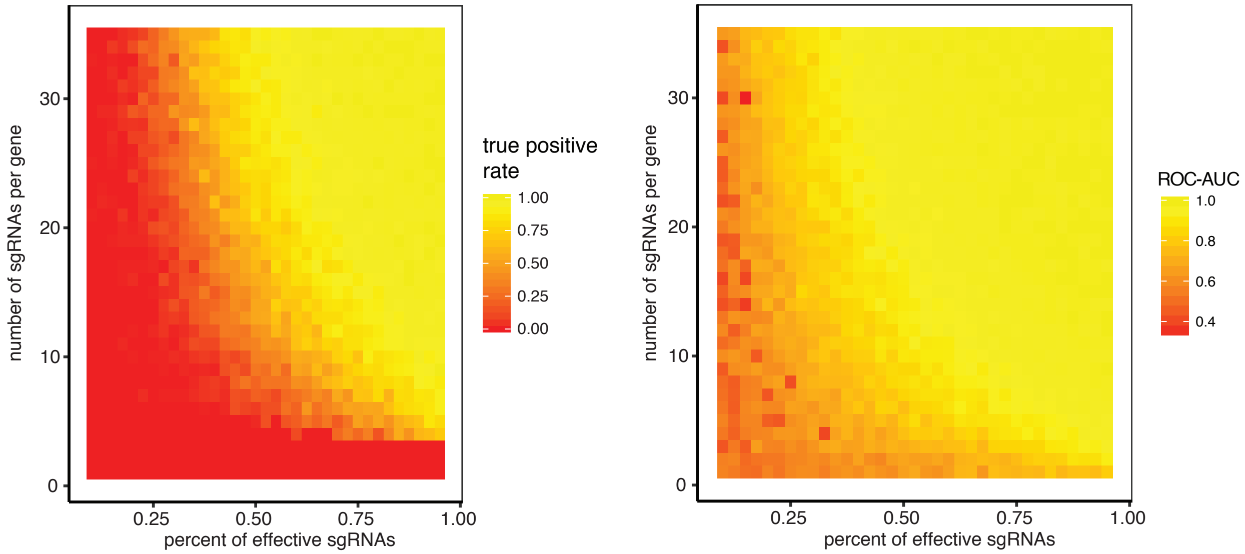

Figure S20: The true positive rate (the percentage of true interesting genes correctly identified) by the Mann-Whitney test at an Benjamani-Hochberg corrected FDR of 0.1 (left) and ROC-AUC (right) for simulated data with varying percent of effective sgRNAs and number of sgRNAs per gene.
